# Data summarizing monitoring and evaluation for three European environmental policies in 9 cases across Europe

**DOI:** 10.1016/j.dib.2019.103785

**Published:** 2019-02-28

**Authors:** Kerry A. Waylen, Kirsty L. Blackstock, Freddy J. van Hulst, Carmen Damian, Ferenc Horváth, Richard K. Johnson, Robert Kanka, Mart Külvik, Christopher J.A. Macleod, Kristian Meissner, Mihaela M. Oprina-Pavelescu, Joan Pino, Eeva Primmer, Geta Rîșnoveanu, Barbora Šatalová, Jari Silander, Jana Špulerová, Monika Suškevičs, Jan Van Uytvanck

**Affiliations:** a Social, Economic & Geographical Sciences, The James Hutton Institute, Cragiebuckler, Scotland, AB15 8QH, UK; b Department of Systems Ecology and Sustainability, University of Bucharest, 91-95 Spl. Independentei, Bucharest, 050095, Romania; c Institute of Ecology and Botany, Centre for Ecological Research, Hungarian Academy of Sciences, Alkotmány u. 2-4, 2163 Vácrátót, Hungary; d Department of Aquatic Sciences and Assessment, Swedish University of Agricultural Sciences, Box 7050, 750 07 Uppsala, Sweden; e Institute of Landscape Ecology of the Slovak Academy of Sciences Stefanikova 3, 814 99 Bratislava, Slovakia; f Institute of Agricultural and Environmental Sciences, Estonian University of Life Sciences, Kreutzwaldi 5, 51006 Tartu, Estonia; g Information and Computational Sciences, The James Hutton Institute, Cragiebuckler, Scotland, AB15 8QH, UK; h Programme for Environmental Information, Finnish Environment Institute - SYKE, Survontie 9a, 40500 Jyväskylä, Finland; i Centre for Research on Ecology and Forestry Applications - CREAF, Universitat Autònoma de Barcelona, E08193 Bellaterra (Cerdanyola del Vallès), Catalonia, Spain; j Freshwater Centre, Freshwater Centre, Finnish Environment Institute - SYKE, P.O. Box 140, 00251 Helsinki, Finland; k Research Institute for Nature and Forest (INBO), Havenlaan 88 bus 73, 1000 Brussels, Belgium

## Abstract

The data presented in this DiB article provide an overview of Monitoring and Evaluation (M&E) carried out for 3 European environmental policies (the Water Framework Directive, the Natura 2000 network of protected areas, and Agri-Environment Schemes implemented under the Common Agricultural Policy), as implemented in 9 cases (Catalonia (Spain), Estonia, Finland, Flanders (Belgium), Hungary, Romania, Slovakia, Scotland (UK), Sweden). These data are derived from reports and documents about monitoring programs that were publicly-available online in 2017. The literature on M&E to support adaptive management structured the issues that have been extracted and summarized. The data is related to the research article entitled “Policy-driven monitoring and evaluation: does it support adaptive management of socio-ecological systems?” [Stem et al., 2005]*.* The information provides a first overview of monitoring and evaluation that has been implemented in response to key European environmental policies. It provides a structured overview that permits a comparison of cases and policies and can assist other scholars and practitioners working on monitoring and evaluation.

**Table:** Specifications table

Subject area	Environmental policy;
More specific subject area	Monitoring; evaluation; European Policy; Water Framework Directive; Natura 2000; Agri-Environment Schemes
Type of data	Tables and text
How data was acquired	Review and analysis of any publicly-available information on monitoring programs
Data format	Summarized, analyzed
Experimental factors	In 2017 the authors searched for publicly available about monitoring programs associated with 3 policy areas: the Water Framework Directive, Natura 2000 and Agri-Environment Schemes under the Common Agricultural Policy. Authors from each organization searched for information about monitoring in the country or region of the organization where they are based: Catalonia (Spain), Estonia, Finland, Flanders (Belgium), Hungary, Romania, Slovakia, Scotland (UK), Sweden. Internet searches of grey and academic literature were used: some authors also contacted policy contacts for advice about where this information could be found, but did not use any information that was not already publicly available.
Experimental features	Bibliographic information on the information sources was recorded (see reference list below), and each author team searched for and summarized information about monitoring and evaluation according to a standard template (see below).
Data source location	Catalonia (Spain), Estonia, Finland, Flanders (Belgium), Hungary, Romania, Slovakia, Scotland (UK), Sweden
Data accessibility	All of the data are within this article.
Related research article	Companion paper to: Waylen, K.A.; Blackstock, K.L.; van Hulst. F.; Damian, C.; Horváth, F.; Johnson, R.; Kanka, R.; Külvik, M.; Macleod, C.; Meissner, C.; Oprina-Pavelescu, M.; Pino, J.; Primmer, E.; Rîșnoveanu, G.; Šatalová, B.; Silander, J.; Špulerová, J.; Suškevičs, M.; Van Uytvanck, J. 2019. Policy-driven monitoring and evaluation: does it support adaptive management of socio-ecological systems? Science of the Total Environment, 662: 373–384 [2]*.*

**Table:** 

**Value of the data** • The data provide the first overview of monitoring and evaluation (M&E) practices carried out by a selection European member states and regions, under 3 European environmental policies (the Water Framework Directive, the Natura 2000 network of protected areas, and Agri-Environment Schemes under the Common Agricultural Policy). • The data permit comparison across cases as well as across policies, and so provide a baseline for comparative studies. • The source of information used to describe monitoring in each case are provided, thus providing a baseline for researchers seeking more in-depth analyses.

## 1 Data

The dataset provided by this article allows an overview of key aspects of monitoring and evaluation carried out in 9 cases in response to 3 European environmental policies. M&E has been identified as an essential part of adaptive management [1]: therefore the information about M&E has been extracted and summarized in terms of attributes that can support adaptive management.

The data are provided in two supplementary files. Appendix A provides a list of the reports and documents from which the data are derived. For ease of reference these lists are separated firstly by each of the 9 cases, and then within each case are subdivided by each policy. Many of the sources are not academic papers, but reports published by government and state agencies: where possible we provide weblinks for ease of access. Appendix B lists of sets of tables summarizing the authors' summaries of aspects of M&E carried out for each policy within each case. Sets of tables describe firstly what is monitored, then describe how monitoring is carried out, and finally describe what is known about how monitoring information is used in evaluation. The summary judgements in these tables are derived from the authors’ review and analysis of the documents provided in Appendix A.

## 2 Experimental design, materials, and methods

In early 2017, nine teams of co-authors agreed to collect information about policy-driven monitoring and evaluation in their country (or in their region, where environmental policy has been devolved). The three European policy areas were: the Water Framework Directive, Natura 2000 network of protected areas, and Agri-Environment Schemes under the Common Agricultural Policy. The nine cases were; Catalonia (Spain), Estonia, Finland, Flanders (Belgium), Hungary, Romania, Slovakia, Scotland (UK), Sweden. In mid-2017 each team used major search engines (e.g. google) to search for any publicly available documentation about monitoring under each policy area in their region or country. To ensure all relevant documents were identified, authors also consulted experts from their networks: however, the study explicitly used only publicly-available documentation, even when participants, their institutions or other experts may have had “insider” or tacit knowledge of the practical implementation of monitoring of some schemes. The final set of documents is contained within the references list. They then documented policy-driven monitoring in their country or region, for all three policy areas, using a common template which is already available as supplementary information to Ref. [2]. The templates were filled in based on information available from publicly available documents, with references to these documents made for all statements within the completed templates. Please see below for a copy of the template which guided the expert review of the documents. The criteria in these table are derived from previously published work on monitoring and evaluation suitable for supporting adaptive management [3].
