# Supplementary material for: Data summarizing monitoring and evaluation for three European environmental policies in 9 cases across Europe
Source: Data Brief. 2019 Feb 28;23:103785. doi: 10.1016/j.dib.2019.103785 (PMC6660552; doi:10.1016/j.dib.2019.103785)
Supplement: Multimedia component 2 [file mmc2.docx]

**Appendix A: List of documents used to analyze policy-driven M&E for each case.**

Information relating to each policy area is listed separately under each geographical case.

Contents

[1. Catalonia (Spain) 2](#_Toc531249149)

[1.1. Agri-environment schemes 2](#_Toc531249150)

[1.2. Natura 2000 2](#_Toc531249151)

[1.3. Water Framework Directive 2](#_Toc531249152)

[2. Estonia 3](#_Toc531249153)

[2.1. Agri-environment schemes 3](#_Toc531249154)

[2.2. Natura 2000 5](#_Toc531249155)

[2.3. Water Framework Directive 5](#_Toc531249156)

[3. Finland 6](#_Toc531249157)

[3.1. Agri-environment schemes 6](#_Toc531249158)

[3.2. Natura 2000 8](#_Toc531249159)

[3.3. Water Framework Directive 9](#_Toc531249160)

[4. Flanders (Belgium) 10](#_Toc531249161)

[4.1. Agri-environment schemes 10](#_Toc531249162)

[4.2. Natura 2000 10](#_Toc531249163)

[4.3. Water Framework Directive 11](#_Toc531249164)

[5. Hungary 11](#_Toc531249165)

[5.1. Agri-environment schemes 11](#_Toc531249166)

[5.2. Natura 2000 12](#_Toc531249167)

[5.3. Water Framework Directive 14](#_Toc531249168)

[6. Romania 15](#_Toc531249169)

[6.1. Agri-environment schemes 15](#_Toc531249170)

[6.2. Natura 2000 17](#_Toc531249171)

[6.3. Water Framework Directive 18](#_Toc531249172)

[7. Scotland (UK) 19](#_Toc531249173)

[7.1. Agri-environment schemes 19](#_Toc531249174)

[7.2. Natura 2000 21](#_Toc531249175)

[7.3. Water Framework Directive 23](#_Toc531249176)

[8. Slovakia 25](#_Toc531249177)

[8.1. Agri-environment schemes 25](#_Toc531249178)

[8.2. Natura 2000 25](#_Toc531249179)

[8.3. Water Framework Directive 26](#_Toc531249180)

[9. Sweden 27](#_Toc531249181)

[9.1. Agri-environment schemes 27](#_Toc531249182)

[9.2. Natura 2000 28](#_Toc531249183)

[9.3. Water Framework Directive 28](#_Toc531249184)

# Catalonia (Spain)

## Agri-environment schemes

1. Catalan Butterfly Monitoring Scheme (2017). "*El Catalan Butterfly Monitoring Scheme o Pla de Seguiment de Ropalòcers de Catalunya.*" The Catalan Butterfly Monitoring Scheme or Monitoring Plan for Ropalócers de Catalunya. Retrieved 24/11/17 from <http://www.catalanbms.org/es/> Published in Catalan.
2. Centre Tecnològic Forestal de Catalunya (CTFC) (no date). "*STEPPE-AHEAD: Steppe-land birds, agriculture practices and economic viability: towards the conservation of threatened species in humanised landscapes.*" Research proposal. Retrieved 24/11/17 from <http://biodiversitat.ctfc.es/ECOLAND/Memories/Fundacions/STEPPE-AHEAD.pdf> Published in English.
3. FEGA (Spanish Agrarian Guarantee Fund) "*SIGPAC (Sistema de Información Geográfica de Parcelas Agrícolas).*" Information on a set of habitat types - this is an interactive map. . Retrieved 24/11/17 from <http://sigpac.mapa.es/fega/visor/> Published in Hungarian.

## Natura 2000

1. BirdLife (no date). "*The Hidden Truth – Spain – Castilla y León - Environmental impact of new Rural Development Programmes – 2014-2020.*" Retrieved 24/11/17 from <http://www.birdlife.org/sites/default/files/attachments/the_hidden_truth_detailed_factsheets_-_spain_cyl.pdf> Published in English.
2. Diputació de Barcelona (no date). "*SITxell,Territorial Analysis.*" The project SITxell (acronym in Catalan for Territorial Information System for the Network of Open Areas in the province of Barcelona), which contains a categorization and assessment of non-urban land based on the analysis of its ecological and socio-economic characteristics. Retrieved 24/11/17 from <http://www.sitxell.eu/en/default.asp> Published in Spanish, Catalan and English.
3. Generalitat de Catalunya - Departament de Territori i Sostenibilitat (2013). "*La xarxa Natura 2000 a Catalunya* " Government of Catalonia - Department of Territory and Sustainability: The Natura 2000 network in Catalonia. Report. . Retrieved 24/11/17 from <http://activarednatura2000.com/wp-content/uploads/2015/08/La-Red-Natura-2000-en-Catalu%C3%B1a.pdf> Published in Catalan.
4. Institució Catalana d’Història Natural (ICHN) (2008). "*Avaluació del sistema d’espais naturals protegits de Catalunya.*" Evaluation of the system of protected natural areas of Catalonia. Retrieved 24/11/17 from <http://ichn.iec.cat/Avaluacio_Espais.htm> Published in Catalan.
5. Institut Català d'Ornitologia (2012). Catalan Institute of Ornithology webiste. Retrieved 24/11/17 from <http://www.ornitologia.org/ca/> Published in Catalan.
6. Ministerio de Agricultura y Pesca, A. y. M. A. (no date). "*Seguimiento y evaluación.*" Monitoring and evaluation - Ministry of Agriculture and Fisheries, Food and Environment. Retrieved 24/11/17 from <http://www.mapama.gob.es/es/biodiversidad/temas/espacios-protegidos/red-natura-2000/rn_cons_seguimiento_evaluacion.aspx> Published in Spanish (site also in Catalan, Galcian, Basque, English and French).
7. Universitat de Barcelona (2015). "*Manual Dels Hàbitats De Catalunya.*" Geobotany Research Group and Cartography of the Vegetation Barcelona University - Manual of Habitats of Catalonia. Retrieved 24/11/17 from <http://www.ub.edu/geoveg/cat/ManualCORINE.php> Published in Spanish, Catalan and English.

## Water Framework Directive

1. Agència Catalana de l'Aigua (2016). "*Jornada: 15 anys de Directiva marc de l’aigua (DMA) Fites assolides i reptes de futur en l’anàlisi de l’estat de les masses d’aigua a Catalunya.*" Workshop: 15 years of WFD Achievements and Challenges: Future in the analysis of the state of water bodies in Catalonia. Retrieved 23/11/17 from <http://www.ub.edu/web/ub/ca/menu_eines/noticies/docs/15dma.pdf> Published in Catalan.
2. Agència Catalana de l'Aigua (2017). "*2013-2018 Monitoring and Control Programme.*" The Monitoring and Control Programme (MCP) is the hydrological planning instrument that defines the mechanisms needed to obtain a coherent and complete overview of the conditions of the bodies of water of the Catalonia River Basin District. Retrieved 23/11/17 from <http://aca-web.gencat.cat/aca/appmanager/aca/aca;jsessionid=83FhZ6QBWcDq9XYm3pnsfz4jNTj2hqfBmVRnNHbQ8vStGrqlB7Xp!-1721432825!795201064?_nfpb=true&_pageLabel=P45800118471380274475241&profileLocale=en> Published in Catalan, Spanish and English.
3. Agència Catalana de l'Aigua (2017). "*Water Agency of Catalan.*" Data a publically avaialable held by Water Agency of Catalan. Retrieved 23/11/17 from <http://aca-web.gencat.cat/aca/appmanager/aca/aca/> Published in Catalan, Spanish and English.
4. Fundación Biodiversidad (2017). "*Estado de Desarrollo de la Red Natura 2000 en España.*" Biodiversity Foundation - Status of development of the Natura 2000 network in Spain. Retrieved 23/11/17 from <http://rednatura2000.wikispaces.com/inicio+estado> Published in Spanish.
5. Munné, A., N. Prat, C. Solà, N. Bonada and M. Rieradevall (2003). "A simple field method for assessing the ecological quality of riparian habitat in rivers and streams: QBR index. ." *Aquatic Conserv: Mar. Freshw. Ecosyst.,*(13): 147–163.

# Estonia

## Agri-environment schemes

1. Avaleht (2016). Rural Economy Research Centre website. Retrieved 28/11/17 from <http://www.maainfo.ee/index.php?page=9&> Published in English and Estonian, .
2. Erametsakeskus (no date). Private Forest Centre. Retrieved 28/11/17 from <http://www.eramets.ee/activities-3/> Published in English, Estonian and Russian.
3. Europa Liit maaelu arenguks (2016). "*Esti maaelu arengukava (MAK) keskkonnaalaste tegevuste püsihindamine.*" The European Union for rural development - A permanent assessment of environmental activities in the Rural Development Plan (MAP). Retrieved 24/11/17 from <http://pmk.agri.ee/mak/avaleht/> Published in Estonian.
4. Europa Liit maaelu arenguks (2016). "*MAK keskkonnaalaste tegevuste püsihindamine.*" The European Union for rural development - Assessment of MAC environmental activities. Retrieved 24/11/17 from <http://pmk.agri.ee/mak/hindamisvaldkonnad/majandus/> Published in Estonian.
5. Europa Liit maaelu arenguks (2016). "*MAK keskkonnaalaste tegevuste püsihindamine - Hindamisvaldkond kompleksuuring mahe– ja tavaviljelusest uuringud.*" The European Union for rural development - Assessment of MAC environmental activities -Field of assessment Complex study on organic and non-organic research. Retrieved 24/11/17 from <http://pmk.agri.ee/mak/hindamisvaldkonnad/kompleksuuring/> Published in Estonian.
6. Maaeluministeerium (2016). "*Eesti maaelu arengukava (MAK) 2014–2020* " Ministry of Rural Affairs - Estonian Rural Development Plan (MAK) 2014-2020. Retrieved 24/11/17 from <https://www.agri.ee/et/eesmargid-tegevused/eesti-maaelu-arengukava-mak-2014-2020> Published in Estonian.
7. Maaeluministeerium (2016). "*Lisa 2. Eesti maaelu arengukava 2014–2020 aasta seirearuande kokkuvõte kodanikele* " Ministry of Rural Affairs - Annex 2. Monitoring Report for the Estonian Rural Development Plan 2014-2020 - Summary of the citizens. Retrieved 24/11/17 from <https://www.agri.ee/sites/default/files/content/arengukavad/mak-2014/mak-2014-2020-seirearuanne-2016-lisa-2.pdf> Published in Estonian.
8. Maaeluministeerium (2017). Ministry of Rural Affairs. Retrieved 24/11/17 from <https://www.agri.ee/et> Published in Estonian.
9. Maaeluministeerium (2017). "*Seire ja hindamine.*" Ministry of Rural Affairs - Monitoring and evaluation. Retrieved 24/11/17 from <https://www.agri.ee/et/eesmargid-tegevused/eesti-maaelu-arengukava-mak-2014-2020/seire-ja-hindamine> Published in Estonian (site also in Russian and English).
10. Maaeluministeerium (2017). "*Seirekomisjoni.*" Ministry of Rural Affairs - The monitoring committee. Retrieved 24/11/17 from <https://www.agri.ee/et/eesmargid-tegevused/eesti-maaelu-arengukava-mak-2014-2020/seire-ja-hindamine/seirekomisjon> Published in Estonian (site also in Russian and English).
11. Maaeluministeerium (2017). "*Vahe- ja järelhindamine.*" Ministry of Rural Affairs - Intermediate and ex post evaluation. Retrieved 24/11/17 from <https://www.agri.ee/et/eesmargid-tegevused/eesti-maaelu-arengukava-mak-2007-2013/seire-ja-hindamine/vahe-ja-jarelhindamine> Published in Estonian (site also in Russian and English).
12. Põllumajandusuuringute Registrite ja Informatsiooni Amet (2015). "*About ARIB.*" Agricultural Research Registers and Information Board - Estonian Agricultural Registers and Information Board (ARIB). Retrieved 24/11/17 from <http://www.pria.ee/en/about> Published in English
13. Põllumajandusuuringute Keskus Saku (2016). "*Eesti Maaelu Arengukava (MAK) Keskkonnamõjuga Meetmete Ekspertgrupi Avakoosolek.*" Agricultural Research Center Saku - Estonian Rural Development Plan (MAK)
14. Environmental Impact Measures Executive Session (slides). Retrieved 24/11/17 from <http://pmk.agri.ee/mak/wp-content/uploads/sites/2/2017/01/4_02_2016_PMK_SH-tagasivaade_IRaa.pdf> Published in Estonian.
15. Põllumajandusuuringute Keskus Saku (no date). "*Eesti Maaelu Arengukava (MAK) Keskkonnamõjuga Meetmete Ekspertgrupi Avakoosolek.*" Agricultural Research Centre website Retrieved 24/11/17 from <http://pmk.agri.ee/> Published in Estonian.
16. Riigi Teataja (2014). "*Eesti maaelu arengukava 2014–2020 seire ja hindamise kord.*" Procedure for monitoring and evaluation of the Estonian Rural Development Plan 2014-2020. Retrieved 28/11/17 from <https://www.riigiteataja.ee/akt/103032017064> Published in English.
17. Riigi Teataja (2014). "*European Union Common Agricultural Policy Implementation Act.*" Retrieved 28/11/17 from <https://www.riigiteataja.ee/en/eli/505122016001/consolide> Published in English.
18. The European Agricultural Fund for Rural Development (2015). "*Eesti maaelu arengukava 2014–2020* " Estonian Rural Development Plan 2014-2020 - report. Retrieved 24/11/17 from <https://www.agri.ee/sites/default/files/content/arengukavad/mak-2014/mak-2014-arengukava-2016-08-11.pdf> Published in Estonian.
19. The European Agricultural Fund for Rural Development (2017). "*Eesti maaelu arengukava 2014–2020* " Estonian Rural Development Plan 2014-2020. Retrieved 24/11/17 from <https://www.agri.ee/sites/default/files/content/arengukavad/mak-2014/mak-2014-arengukava-2016-08-11.pdf> Published in Estonian.
20. The European Agricultural Fund for Rural Development (2017). "*Iga-aastane rakendusaruanne Estonia - Rural Development Programme (National).*" Annual Implementation Report. Retrieved 24/11/17 from <https://www.agri.ee/sites/default/files/content/arengukavad/mak-2014/mak-2014-2020-seirearuanne-2016-rakendusaruanne.pdf> Published in Estonian.

## Natura 2000

1. Estonian Environment Agency (2014). "*Estonian Environmental Indicators – development and outcomes.*" Report. Retrieved 28/11/17 from <http://www.keskkonnainfo.ee/failid/indikaatorid_ENG_web.pdf> Published in English.
2. Keskkonnaagentuur (2008). "*Estonian environmental indicators 2009.*" Estonian Environment Agency - Publications & Reviews Retrieved 15/11/17 from <http://www.keskkonnainfo.ee/main/index.php/en/component/content/article/420> Published in English (and Estonian).
3. Keskkonnaagentuur (2008). "*Keskonnainfo.*" Information portal for information systems managed by the Environment Agency. Annual monitoring reports are publically available. Retrieved 15/11/17 from <https://sso.keskkonnainfo.ee> Published in Estonian.
4. Keskkonnaagentuur (2008). "*Väljaanded & ülevaated.*" Estonian Environment Agency - Publications & Reviews Retrieved 15/11/17 from <http://www.keskkonnaagentuur.ee/et/keskkonnaseirevaljaanded> Published in Estonian.
5. Keskkonnaseire Infosüsteem (no date). Environmental Monitoring Information System (accessible through Estonian ID identification only). Retrieved 28/11/17 from <https://kese.envir.ee/kese/> Published in Estonian.
6. Riigi Teataja (2017). "*Environmental Monitoring Act.*" Retrieved 28/11/17 from <https://www.riigiteataja.ee/en/eli/ee/Riigikogu/act/504092017009/consolide> Published in English.
7. Riigi Teataja (2017). "*Kaitsekorralduskava koostamise ja kinnitamise kord ning kaitsekorralduskava kinnitaja määramine.*" Establishment and validation of a management plan and designation of a manager of the management plan. Retrieved 28/11/17 from <https://www.riigiteataja.ee/akt/13228916?leiaKehtiv> Published in Estonian.

## Water Framework Directive

1. Keskkonnaagentuur (2008). Estonian Environmental Agency website. Retrieved 15/11/17 from <http://www.keskkonnaagentuur.ee/> Published in Estonian (site also in English).
2. Keskkonnaagentuur (2008). "*Keskonnainfo.*" Information portal for information systems managed by the Environment Agency. Annual monitoring reports are publically available. Retrieved 15/11/17 from <https://sso.keskkonnainfo.ee> Published in Estonian.
3. Keskkonnaministeerium (2016). "*Inimtegevuse mõju vesikonnas.*" Ministry of the Environment - Impact of human activity on river basin. Retrieved 28/11/17 from <http://www.envir.ee/et/vesi/veemajanduskavad/veemajanduskava-2015-2021/inimtegevuse-moju-vesikonnas> Published in Estonian (site also in English and Russian).
4. Keskkonnaministeerium (2016). "*Keskkonnaseire.*" Ministry of the Environment - Environmental monitoring. Retrieved 28/11/17 from <http://www.envir.ee/et/keskkonnaseire> Published in Estonian (site also in English and Russian).
5. Keskkonnaministeerium (2016). "*Veemajanduskavad 2009-2015.*" Ministry of the Environment - Water Management Schemes 2009-2015. Retrieved 28/11/17 from <http://www.envir.ee/et/veemajanduskavad-2009-2015> Published in Estonian (site also in English and Russian).
6. Keskkonnaministeerium (2016). "*Veemajanduskavad 2015-2021.*" Ministry of the Environment - Water Management Schemes 2015-2021. Retrieved 28/11/17 from <http://www.envir.ee/et/veemajanduskavad-2009-2015> Published in Estonian (site also in English and Russian).
7. Keskkonnaministeerium (2016). "*Veepoliitika raamdirektiivi rakendamine.*" Ministry of the Environment - Implementation of the Water Framework Directive. Retrieved 28/11/17 from <http://www.envir.ee/et/veepoliitika-raamdirektiivi-rakendamine> Published in Estonian (site also in English and Russian).
8. Keskkonnaministeerium (2017). "*Veemajanduskavad - Veemajanduskavade koostamine ja ajakohastamine.*" Ministry of the Environment - Water management plans - Preparation and updating of water management plans. Retrieved 28/11/17 from <http://www.envir.ee/et/veemajanduskavad> Published in Estonian (site also in English and Russian).
9. Keskkonnaministeeriumi veeosakond (no date). "*Veeseire Ümberkorraldamine Veepoliitika Raamdirektiivi Nõuetele Vastavate Seireprogrammide Koostamine.*" Water Department of the Ministry of the Environment - Waterwear restoration compliance with the requirements of the water policy framework construction of monitoring programs. Report. Retrieved 28/11/17 from <https://www.envir.ee/sites/default/files/uusseire.pdf> Published in Estonian.
10. Riigi Teataja (1994). "*Water Act.*" Retrieved 28/11/17 from <https://www.riigiteataja.ee/en/eli/520062017004/consolide> Published in English.
11. Riigi Teataja (2016). "*Nõuded vesikonna veeseireprogrammide kohta.*" Requirements for river basin water monitoring programs. Retrieved 287/11/7 from <https://www.riigiteataja.ee/akt/112042011009> Published in Estonian.

# Finland

## Agri-environment schemes

1. Aakkula, J. and J. Leppänen (2014). "*Follow-up study on the impacts of agri-environment measures (mytvas 3) – Final report.*" Retrieved 15/11/17 from <http://mmm.fi/documents/1410837/1720628/MMM_mytvas_loppuraportti_WEB.pdf/2cc8f041-82f2-4bbf-85e3-bd4a8d6964b3> Published in Finnish.
2. Antikainen, J., P. Kahila, S. Palviainen, S. Pyykkönen and M. Yli-Koski (2014). "*Manner-Suomen Maaseudun Kehittämisohjelman 2014–2020 Ennakkoarviointi.*" Pre-evaluation of the Rural Development Program of Mainland Finland 2014-2020. Retrieved 15/11/17 from <http://mmm.fi/documents/1410837/1720628/MMM_M-S_ennakkoarviointi_22014_web.pdf/4b2352ae-a68a-408b-8489-a8935a432117> Published in Finnish.
3. Biodiversity.fi (no date). "*FA10 Farmland butterflies.*" Report on butterfly population monitoring and data. Retrieved 20/11/17 from <https://www.biodiversity.fi/en/habitats/farmlands/fa10-farmland-butterflies> Published in English and Finnish.
4. Ekroos, J., J. Heliölä and M. Kuussaari (2010). "Homogenization of lepidopteran communities in intensively cultivated agricultural landscapes." *Journal of Applied Ecology* 47(2): 459-467. <http://dx.doi.org/10.1111/j.1365-2664.2009.01767.x>.
5. European Commission (2014). "*Factsheet on 2014-2020 Rural Development Programme for Mainland Finland* ". Retrieved 15/11/17 from <https://ec.europa.eu/agriculture/sites/agriculture/files/rural-development-2014-2020/country-files/fi/factsheet-mainland_en.pdf> Published in English.
6. Finland's Environmental Administration (2017). "*Monitoring butterflies in Finnish agricultural landscapes.*" Details of a butterfly monitoring network, methods, sites and results. . Retrieved 20/11/17 from <http://www.ymparisto.fi/en-US/Nature/Species/Species_monitoring/Monitoring_butterflies_in_Finnish_agricultural_landscapes> Published in English and Finnish.
7. Finland's Environmental Administration (2017). "*Monitoring butterflies in Finnish agricultural landscapes.*" Details of a butterfly monitoring network, methods, sites and results. . Retrieved 20/11/17 from <http://www.ymparisto.fi/en-US/Nature/Species/Species_monitoring/Monitoring_butterflies_in_Finnish_agricultural_landscapes> Published in English or Finnish.
8. Grammatikopoulou, I., E. Pouta, M. Salmiovirta and K. Soini (2012.). "Heterogeneous preferences for agricultural landscape improvements in southern Finland. ." *Landscape and Urban Planning*: 107: 181ern F.
9. Kotmäki, N. (2015). "Näytteenottotiheyden vaikutus tila-arvion tarkkuuteen. [The effect of sampling frequency on the accuracy of the status estimate. Report of Finnish Environment Institute]." 1.6. Retrieved 20/11/17 from <http://www.syke.fi/download/noname/%7BDBADBB67-4B0A-4923-A04E-77E796A5AE59%7D/118998>. Published in Finnish.
10. Kuussaari, M., J. Heliola, J. Tiainen and J. Helenius (2008. ). "Maatalouden ymparistotuen merkitys luonnon monimuotoisuudelle ja maisemalle: MYTVAS-loppuraportti 2000–2006 [Importance of agro-environmental support for biodiversity and landscape: MYTVAS Final Report 2000-2006]." *Suomen ymparisto 4/2008.*(Helsinki: Suomen ymparistokeskus (SYKE).): 208 s.
11. Maa- ja metsätalousministeriö (2014). "*Maatalouden ympäristötuen vaikuttavuuden seurantatutkimus.*" Ministry of Agriculture and Forestry: Follow-up survey on the effectiveness of agri-environment support. Retrieved 20/11/17 from <http://mmm.fi/mytvas> Published in Finnish, English and Swedish.
12. Mäkelä-Kurtto, R. and J. Sippola (2008). "Monitoring of Finnish arable land: changes in soil quality between 1987 and 1998." *Agricultural and Food Science* 11(4): 273-284. <https://journal.fi/afs/article/view/5730>.
13. Ministry of Environment (no date). "*Welcome to Biodiversity.fi.*" Biodiversity.fi includes more than 110 indicators reflecting the state and development of various components of biological diversity as well as factors driving changes in Finland's nature. Retrieved 20/11/17 from <https://www.biodiversity.fi/en/home> Published in English and Finnish.
14. Oikeusministeriö Justitieministeriet (2010). "*Valtioneuvoston asetus vesiympäristölle vaarallisista ja haitallisista aineista annetun valtioneuvoston asetuksen muuttamisesta.*" Ministry of Justice: Amendments to the Government Decree on Hazardous and Noxious Substances - includes statutory advice on priority substances and other pollutants. Retrieved 20/11/17 from <http://www.finlex.fi/fi/laki/alkup/2010/20100868> Published in Finnish.
15. Opendata.fi (2015). "*Suomen avoimen datan palvelu.*" Finland's open data service. Retrieved 20/11/17 from <https://www.opendata.fi/en> Published in English, Finnish and Swedish.
16. Pyykkonen, P., S. Backman and E. Puttaa (2013). "Rakennemuutos Suomen kotielaintaloudessa. [Structural change in the Finnish housing economy.]." *PTT työpapereita 143.* Finnish with an English abstract.
17. Pyykkonen, P., H. Lehtonen and A. Koivisto (2010). "Maatalouden rakennekehitys ja investointitarve vuoteen 2020. [Agricultural structural development and investment needs by 2020.] " *Maataloudeniculture 2010-2020* Finnish with an English abstract: 2SBN 978-952-224-061-973 (pdf), ISSN 1796-4784 (pdf). Retrieved 20/11/17 from <http://www.ptt.fi/dokumentit/tp1125_1111100930.pdf>.
18. Rääpysjärvi, J., H. Hämäläinen and J. Aroviita (2016). "Macrophytes in boreal streams: Characterizing and predicting native occurrence and abundance to assess human impact." *Ecological indicators* 64(In English): 309–318.
19. Ruuhijärvi, J., T. Sutela and M. Olin (2014). "*Maa- ja metsätalouden vesistövaikutukset –seminaari Helsinki 9.5.2014: Maa- ja metsätalouden kuormituksen vaikutukset kalastoon.*" Seminar on water spill in agriculture and forestry: Land and forestry load effects on fish. Retrieved 24/11/17 from <http://www.syke.fi/hankkeet/maamet>. Published in Finnish.
20. Soini, K. (2007). "Beyond the ecological hot spots : understanding local residentsogical hot spots : understanding agricultural landscapes. ." *Turun yliopiston julkaisuja. Sarja A [Publications of the University of Turku]* Diss. 206(102): 11 (in Finnish).
21. Suomen ympäristökeskus (2015). "*Maatalouden ympäristötuen vaikuttavuuden seurantatutkimus (MYTVAS3).*" Finnish Environment Institute: Monitoring of the Effectiveness of Agri-environmental Support. Retrieved 20/11/17 from <http://www.syke.fi/fi-FI/Tutkimus__kehittaminen/Tutkimus_ja_kehittamishankkeet/Hankkeet/Maatalouden_ymparistotuen_vaikuttavuuden_seurantatutkimus_MYTVAS3/Maatalouden_ymparistotuen_vaikuttavuuden%282397%29> Published in Finnish.

## Natura 2000

1. BirdLife Suomi (2017). "*BirdLife Finland.*" Partner of BirdLife International and parent organization of 30 Finnish regional ornithological societies. Collects birdwatchers’ observations. Retrieved 20/11/17 from <https://www.birdlife.fi/in-english/> Published in English, Finnish, Swedish.
2. Centre for Economic Development Transport and the Environment (2016). "*The Centres for Economic Development, Transport and the Environment (ELY Centres).*" Centre responsible for the regional implementation and development tasks of the central government. It monitors aquatic habitats. Retrieved 20/11/17 from <https://www.ely-keskus.fi/en/web/ely-en/> Published in Finnish, English, Estonian, Russian, Swedish.
3. Finnish Environment Institute (no date). "*SKYE* " The institute and institute website reports to the Commission; Supports monitoring and research; Maintains species database: Hertta used in land-use planning and permit systems; Hosts the information portal; Conducts butterfly monitoring. Retrieved 20/11/17 from <http://www.syke.fi/en-US> Published in English, Finnish and Swedish.
4. LAJI.fi (no date). "*Suomen Lajitietokeskus,.*" The Finnish Information Center collects and combines Finnish species information. . Retrieved 20/11/17 from <https://www.laji.fi/about> Published in Finnish.
5. LOUMUS: Finnish Museum of Natural History (no date). "*Finnish Museum of Natural History.*" Maintains collections and hosts the Finnish Biodiversity Information Facility. Retrieved 20/11/17 from <https://www.luomus.fi/en> Published in English, Finnish, Swedish and Russian.
6. LUKE: Natural Resources Institute Finland (2016). "*Natural Resources Institute Finland.*" Institute conducts forest, game and fish inventories. . Retrieved 20/11/17 from <https://www.luke.fi/en/> Published in English, Finnish and Swedish.
7. Metsähallitus (2017). "*Finnish body managing state land and protected areas.*" A state-owned enterprise, responsible for the management of one third of Finland’s surface area. They aim to use and develop state-owned land and water areas responsibly, in a way that maximises their benefits to society as a whole. It also monitors the status of Directive habitats in protected areas. . Retrieved 20/11/17 from <http://www.metsa.fi/web/en> Published in English, Finish and Swedish
8. Ympäristöministeriö (1996 [translated 1997]). "*Nature Conservation Act.*" Ministry of Environment: Act amended in 2011. Retrieved 20/11/17 from <http://www.finlex.fi/en/laki/kaannokset/1996/en19961096> Published in English (and Finnish).
9. Ympäristöministeriö (1997). "*Nature Conservation Decree (160/1997).*" Ministry of Environment: Lists protected species, threatened species, species needing special protection, and species that need strict protection according to the EU Habitats Directive.

## Water Framework Directive

1. Aroviita, J., S. Hellsten, J. Jyväsjärvi, L. Järvenpää, M. Järvinen, S. M. Karjalainen, P. Kauppila, A. Keto, M. Kuoppala, K. Manni, J. Mannio, S. Mitikka, M. Olin, J. Perus, A. Pilke, M. Rask, J. Riihimäki, A. Ruuskanen, K. Siimes, T. Sutela, T. Vehanen and K.-M. Vuori (2012). "*Ohje pintavesien ekologisen ja kemiallisen tilan luokitteluun vuosille 2012–2013 − päivitetyt arviointiperusteet ja niiden soveltaminen.*" Finnish Environment Institute - Guidance on ecological and water surface waters chemical status classification for 2012-2013: Updated criteria and their application. Retrieved 20/11/17 from <https://helda.helsinki.fi/bitstream/handle/10138/41788/OH_7_2012.pdf?sequence=6> Published in Finnish.
2. Finnish Environment Institute (SYKE) (2017). "*Avoin tieto.*" SYKE provides open data and information for building a sustainable environment and society. Users have to log in to access data. Retrieved 20/11/17 from <http://www.syke.fi/avointieto> Published in Finnish.
3. Granlund, K., Räike, A.,, P. Ekholm, K. Rankinen and S. Rekolainen (2005). "Assessment of water protection targets for agricultural nutrient loading in Finland." *Journal of Hydrology* 304(1): 251-260. .
4. Karonen, M., A. Mäntykoski, E. Nylander and K. Lehto (2015). "*Vesien tila hyväksi yhdessä - Kymijoen-Suomenlahden vesienhoitoalueen vesienhoitosuunnitelma vuosiksi 2016–2021.*" The state of waters together - The water management area of the Kymijoki Gulf of Finland water management plan for 2016-2021. Retrieved 24/11/17 from <http://www.doria.fi/bitstream/handle/10024/121868/Raportteja%20132%202015.pdf?sequence=2> Published in Finnish.
5. Kauppila, J. (2016). "*Vesienhoitosuunnitelman oikeudellisen vaikuttavuuden rakentuminen.*" The legal effectiveness of the river basin management plan. Dissertations in Social Sciences and Business Studies, no 138. University of Eastern Finland. . Retrieved 21/11/17 from <http://epublications.uef.fi/pub/urn_isbn_978-952-61-2309-7/urn_isbn_978-952-61-2309-7.pdf> Published in Finnish (abstract in English).
6. Mäkelä-Kurtto, R. and J. Sippola (2008). "Monitoring of Finnish arable land: changes in soil quality between 1987 and 1998." *Agricultural and Food Science*(4): 273-284%V 211. <https://journal.fi/afs/article/view/5730>.
7. Meissner, K., J. Aroviita, S. Hellsten, M. Järvinen, S. M. Karjalainen, M. Kuoppala, H. Mykrä and K.-M. Vuori (2016). "*Jokien Ja Järvien Biologinen Seuranta – Näytteenotosta Tiedon Tallentamiseen.*" Biological monitoring paper. Gives guidance on relevant sampling methods. Retrieved 20/11/17 from <http://www.ymparisto.fi/download/noname/%7BB948034F-7F9D-4EAB-A153-92FA2DDEDBBE%7D/29725> Published in Finnish.
8. Oikeusministeriön (2004). "*Laki vesienhoidon ja merenhoidon järjestämisestä.*" Ministry of Justice: Law on the organization of water management and seafaring. Retrieved 20/11/17 from <http://finlex.fi/fi/laki/ajantasa/2004/20041299?search%5Btype%5D=pika&search%5Bpika%5D=1299> Published in Finnish with English version at http://finlex.fi/en/laki/kaannokset/2004/en20041299?search%5Btype%5D=pika&search%5Bpika%5D=water).
9. Seppälä, J., M. Melanen, T. Jouttijärvi, L. Kauppi and N. Leikola (1998). "Forest industry and the environment: a life cycle assessment study from Finland." *Resources, Conservation and Recycling* 23(1): 87-105.
10. Velthof, G. L., J. P. Lesschen, J. Webb, S. Pietrzak, Z. Miatkowski, M. Pinto and O. Oenema (2014). "The impact of the Nitrates Directive on nitrogen emissions from agriculture in the EU-27 during 2000–2008." *Science of the Total Environment,*(468): 1225-1233.

# Flanders (Belgium)

## Agri-environment schemes

1. Instituut Natuur - En Bosonderzoek (2017). "*De Biologische Waarderingskaart (BWK).*" Institute of Nature and Forest Research - The Biological Valuation Map (BWK). Retrieved 28/11/17 from <https://www.inbo.be/nl/de-biologische-waarderingskaart-bwk> Published in Dutch (site also in English).
2. Natuurpunt (2017). "*Meetnetten.*" Nature Point - Measurement networks. Retrieved 28/11/17 from <https://www.natuurpunt.be/pagina/meetnetten> Published in Dutch.
3. Natuurpunt (2017). "*Waarnemingen.*" The observation website of the biggest nature conservation NGO in Flanders (Natuurpunt), containing thousands of records of all kinds of organisms. . Retrieved 28/11/17 from <https://waarnemingen.be/index.php> Published in Dutch (and many other European languages).
4. Vlaamse Land Maatschappij (2017). "*Beheerovereenkomsten.*" Flemish Land Company - Management agreements. Retrieved 28/11/17 from <https://www.vlm.be/nl/themas/beheerovereenkomsten#anker1> Published in Dutch (site also in English, French and German).
5. Vlaanderen Departement Landbouw En Visserij (no date). "*Publicaties.*" Flanders Department of Agriculture and Fisheries - Publications. Retrieved 28/11/17 from <http://lv.vlaanderen.be/nl/landbouwbeleid/plattelandsontwikkeling/publicaties#Brochure> Published in Dutch.

## Natura 2000

1. Agentschap Natuur en Bos (2017). "*Soortenbescherming.*" Agency for Nature and Forestry - Species protection. Retrieved 28/11/17 from <https://www.natuurenbos.be/SBP> Published in Dutch**:** 28/11/17.
2. Instituut Natuur - En Bosonderzoek (2017). Midlevel reports are available on the website of INBO (Research Institute for Nature and Forest). Retrieved 28/11/17 from <https://www.inbo.be/> Published in Dutch.
3. Instituut Natuur - En Bosonderzoek (2017). "*Natura 2000 - Programmatische Aanpak Stikstof (PAS).*" Institute of Nature and Forest Research - Programmatic Approach to Nitrogen (PAS). Retrieved 28/11/17 from <https://www.inbo.be/nl/de-biologische-waarderingskaart-bwk> Published in Dutch.
4. Louette, G., D. Adriaens, G. De Knijf and D. Paelinckx (2013). "Staat van instandhouding (status en trends) habitattypen en soorten van de Habitatrichtlijn (rapportageperiode 2007-2012). Rapporten van het Instituut voor Natuur- en Bosonderzoek 2013 (INBO.R.2013.23)." *[State of conservation (status and trends) habitat types and species of the Habitats Directive (reporting period 2007-2012). Reports from the Institute for Nature and Forest Research 2013]* Instituut voor Natuur- en Bosonderzoek, Brussel
5. Paelinckx, D., K. Sannen, V. Goethals, G. Louette, J. Rutten and M. Hoffmann (2009). "*Gewestelijke doelstellingen voor de habitats en soorten van de Europese Habitat- en Vogelrichtlijn voor Vlaanderen.*" Regional objectives for the habitats and species of the European Habitats and Birds Directives for Flanders. Announcements from the Institute for Nature and Forest Research. Retrieved 21/11/17 from <https://purews.inbo.be/ws/files/5493595/Paelinckx_etal_2009_GewestelijkeDoelstellingenHabitatsSoortenEuropeseHabitatVogelrichlijnVlaanderen.pdf> Published in Dutch**:** 16-45.

## Water Framework Directive

1. Coördinatiecommissie Integraal Waterbeleid (no date). "*Bekkens.*" Coordination Committee on Integrated Water Policy - Information about River Basins. Retrieved 28/11/17 from <http://www.integraalwaterbeleid.be/nl/bekkens/> Published in Dutch (site in English and French).
2. Coördinatiecommissie Integraal Waterbeleid (no date). "*Watertoets.*" Coordination Committee on Integrated Water Policy - Water test. Retrieved 28/11/17 from <http://www.integraalwaterbeleid.be/nl/beleidsinstrumenten/watertoets> Published in Dutch (English and French).
3. Geopunt (no date). "*Kaart.*" Interactive database map. Retrieved 28/11/17 from <http://www.geopunt.be/> Published in Dutch.
4. Vismigratie (2017). Interactive data map of fish migration monitoring, includes context items such as habitation, roads, adjacent land use. Retrieved 28/11/17 from <http://vismigratie.vmm.be/vismigratie/> Published in Dutch.
5. Vlaamse Milieumaatscappij (2015). "*Evaluatie saneringsinfrastructuur 2015.*" Flemish Environment Agency - Evaluation of the remediation infrastructure 2015. Retrieved 28/11/17 from <https://www.vmm.be/publicaties/evaluatie-saneringsinfrastructuur-2015> Published in Dutch.
6. Vlaamse Overheid (2017). "*Integraal Waterbeleid Ijzerbekken.*" Flemish Government site on Integral Water Policy. Retrieved 28/11/17 from <http://www.integraalwaterbeleid.be/nl/bekkens/ijzerbekken/in-de-kijker/juni-2017-gebiedsgerichte-en-integrale-aanpak-voor-de-blankaart> Published in Dutch (site in English and French).
7. Vlaamse Overheid (2017). "*Mira Milieurapport Vlaanderen Vlaamse Milieumaatschappij.*" Flemish Government - Mira Environmental Report Flanders Flemish Environment Agency. Retrieved 28/11/17 from <http://www.milieurapport.be/nl/feitencijfers/milieuthemas> Published in Dutch (site in English and French).

# Hungary

## Agri-environment schemes

1. Babai, D., A. Tóth, I. Szentirmai, M. Biró, A. Máté, L. Demeter, M. Szépligeti, A. Varga, Á. Molnár, R. Kun and Z. Molnár (2015). "Do conservation and agri-environmental regulations effectively support traditional small-scale farming in East-Central European cultural landscapes?" *Biodiversity and Conservation* 24(13): 3305-3327. <https://doi.org/10.1007/s10531-015-0971-z>.
2. Inger, R., R. Gregory, J. P. Duffy, I. Stott, P. Voříšek and K. J. Gaston (2015). "Common European birds are declining rapidly while less abundant species' numbers are rising." *Ecology Letters* 18(1): 28-36. <http://dx.doi.org/10.1111/ele.12387>.
3. Körtáj, T. I. K. (2013). "Az Új Magyarország Vidékfejlesztési Program (ÚMVP) környezeti eredmény- és 4 hatásindikátora értékeinek a meghatározása (New Hungary Rural Development Program (ÚMVP) environment result and 4 impact indicators)." *Pilisszentlászló,*: 285
4. Kovács-Hostyánszki, A. and A. Báldi (2012). "Set-aside fields in agri-environment schemes can replace the market-driven abolishment of fallows." *Biological Conservation* 152(Supplement C): 196-203. <http://www.sciencedirect.com/science/article/pii/S0006320712001826>.
5. Mihók, B., M. Biró, Z. Molnár, E. Kovács, J. Bölöni, T. Erős, T. Standovár, P. Török, G. Csorba, K. Margóczi and A. Báldi (2017). "Biodiversity on the waves of history: Conservation in a changing social and institutional environment in Hungary, a post-soviet EU member state." *Biological Conservation* 211(Part A): 67-75. <http://www.sciencedirect.com/science/article/pii/S0006320717307875>.
6. Mihók, B., E. Kovács, B. Balázs, G. Pataki, A. Ambrus, D. Bartha, Z. Czirák, S. Csányi, P. Csépányi, M. Csőszi, G. Dudás, C. Egri, T. Erős, S. Gőri, G. Halmos, A. Kopek, K. Margóczi, G. Miklay, L. Milon, L. Podmaniczky, J. Sárvári, A. Schmidt, K. Sipos, V. Siposs, T. Standovár, C. Szigetvári, L. Szemethy, B. Tóth, L. Tóth, P. Tóth, K. Török, P. Török, C. Vadász, I. Varga, W. J. Sutherland and A. Báldi (2015). "Bridging the research-practice gap: Conservation research priorities in a Central and Eastern European country." *Journal for Nature Conservation* 28(Supplement C): 133-148. <http://www.sciencedirect.com/science/article/pii/S1617138115300236>.
7. Nébih (no date). "*Erdőtérkép.*" The National Forest Management Database. Retrieved 23/11/17 from <http://erdoterkep.nebih.gov.hu/> Published in Hungarian.
8. Nébih (no date). "*Introduction of the National Food Chain Safety Office (NFCSO).*" Retrieved 22/11/17 from <https://www.nebih.gov.hu/en/> Published in English.
9. The National Game Management Database (2017). Webpage. Retrieved 23/11/17 from <http://ova.info.hu/index-en.html> Published in English and Hungarian.

## Natura 2000

1. A magyar állami természetvédelem (2005). "*Mintavételi módszerek* " Sampling methods. Monitoring protocols are described and downloadable here for various monitoring activities from the National Biodiversity Monitoring System (NBmR). . Retrieved 22/11/17 from <http://www.termeszetvedelem.hu/index.php?pg=sub_472> Published in Hungarian.
2. A magyar állami természetvédelem (2005). "*Nemzeti Biodiverzitás-monitorozó Rendszer (NBmR)* " Hungarian State Nature Conservation - National Biodiversity Monitoring System (NBmR). Retrieved 22/11/17 from <http://www.termeszetvedelem.hu/nbmr> Published in Hungarian.
3. A magyar állami természetvédelem (2012). "*TIR kezdőlap.*" Hungarian State Nature Conservation - central database of TIR Retrieved 22/11/17 from <http://www.termeszetvedelem.hu/tir> Published in Hungarian.
4. Horváth, F. (2006). Élőhely-térképezés: élőhelyek mintázata és változása a tájban [Habitat mapping: Habitat patterns and changes in the landscape ]. *Élőhelyek, mohák és gombák.[Habitats, Bryophytes and Fungi] in Hungarian with English summary, Környezetvédelmi és Vízügyi Minisztérium, Természetvédelmi Hivatal,*. K. Török and L. Fodor**:** 17-28.
5. Kárpáti Erdeink Kutatása "*A Projektről.*" Carpathian Forest Research - The multipurpose state assessment underlying the protection of forest communities in the Hungarian Carpathians. Retrieved 22/11/17 from <http://karpatierdeink.hu/hun/a-projektrol> Published in Hungarian and English.
6. Körtáj, T. I. K. (2013). "*Az Új Magyarország Vidékfejlesztési Program (ÚMVP) környezeti eredmény- és 4 hatásindikátora értékeinek a meghatározása, Final Report, Pilisszentlászló.*" Definition of the environmental performance and 4 impact indicators of the New Hungary Rural Development Program (ÚMVP). Retrieved 23/11/17 Published in Hungarian**:** 285.
7. Magyar Madártan és Természetvédelmi Egyesület (2016). "*SH/4/8 projekt Sustainable Conservation on Natura 2000 sites in Hungary Project Closing Conference.*" Hungarian Natura 2000 Project blog post. . Retrieved 24/11/17 from <http://www.natura.2000.hu/en/groups/project-blog> Published in Hungarian and English.
8. Magyar Madártan és Természetvédelmi Egyesület (no date). "*Madármonitoring.*" Bird monitoring - MME Monitoring Centre. Retrieved 22/11/17 from <http://www.mme.hu/madarmonitoring> Published in Hugarian.
9. Magyar Madártan és Természetvédelmi Egyesület (no date). "*Mindennapi Madaraink Monitoringja (MMM).*" Everyday bird monitoring - Hungarian Bird and Nature Conservation Association. Retrieved 22/11/17 from <http://www.mme.hu/mindennapi-madaraink-monitoringja-mmm> Published in Hugarian.
10. Magyar Madártan és Természetvédelmi Egyesület (no date). "*MMM adatbázis.*" Database developed by the Hungarian Association of Birds and Nature Conservation (MME). Retrieved 22/11/17 from <http://www.mme.hu/szervezet/monitoring-kozpont> Published in Hugarian.
11. Magyar Madártan és Természetvédelmi Egyesület (no date). "*Monitoring Központ.*" Monitoring Centre - Hungarian Bird and Nature Conservation Association. Retrieved 22/11/17 from <http://www.mme.hu/szervezet/monitoring-kozpont> Published in Hugarian.
12. Magyar Madártan és Természetvédelmi Egyesület (no date). "*Ritka és Telepesen fészkelő madarak Monitoringja (RTM).*" RTM - scheme to monitor rare and colonial birds - Hungarian Bird and Nature Conservation Association. Retrieved 22/11/17 from <http://www.mme.hu/ritka-es-telepesen-feszkelo-madarak-monitoringja-rtm> Published in Hugarian.
13. Magyar Madártan és Természetvédelmi Egyesület (no date). "*Vonuló Vízimadár Monitoring program.*" VVM – monitoring of migrating waterfowl birds - Hungarian Bird and Nature Conservation Association. Retrieved 22/11/17 from <http://vizimadaradatbazis.mme.hu/page/programme> Published in Hugarian.
14. Magyarország Kormánya (2016). "*Evaluation.*" Hungarian Government - The National Rural Development Plan (NVT), the New Hungary Rural Development Programme (ÚMVT) and the recently developed Rural Development Programme (VP) went under social agreements/evaluation. . Retrieved 23/11/17 from <https://www.palyazat.gov.hu/evaluation> Published in English and Hungarian.
15. Magyarország Kormánya (2016). "*Természetvédelmi Stratégiai Projekt indul a Földművelésügyi Minisztériumban.*" "Strategic Nature Conservation Project starts at the Ministry of Agriculture" A new project started in 2016 to support nature conservation policy by improved knowledge base (NATURA) and assessment procedures. Retrieved 22/11/17 from <http://www.kormany.hu/hu/foldmuvelesugyi-miniszterium/kornyezetugyert-agrarfejlesztesert-es-hungarikumokert-felelos-allamtitkarsag/hirek/termeszetvedelmi-strategiai-projekt-indul-a-foldmuvelesugyi-miniszteriumban> Published in Hungarian.
16. Mihók, B., M. Biró, Z. Molnár, E. Kovács, J. Bölöni, T. Erős, T. Standovár, P. Török, G. Csorba, K. Margóczi and A. Báldi (2017). "Biodiversity on the waves of history: Conservation in a changing social and institutional environment in Hungary, a post-soviet EU member state." *Biological Conservation* 211(Part A): 67-75. <http://www.sciencedirect.com/science/article/pii/S0006320717307875>.
17. MTA Centre for Ecological Research (2016). "*Á-NÉR 2011 habitats, maps and descriptions.*" Retrieved 22/11/17 from <http://www.novenyzetiterkep.hu/english/node/1090> Published in Hugarian and English.
18. Nagy, S., K. Nagy and T. Szép (2009). "Potential Impact of EU Accession on Common Farmland Bird Populations in Hungary." *Acta Ornithologica* 44(1): 37-44.
19. Szegleti, Z., G. Csicsek, G. Szabó, Z. Zimmermann, J. Bölöni and H. F. (2017). "Erdőtermészetesség szempontú értékelési módszer a Pannon életföldrajzi régió Natura 2000 erdei élőhelytípusainak szerkezete és funkció monitorozása alapján [Forest naturalness based evaluation method for assessment of Natura 2000 forests of the Pannonian region to monitor structure and function]." *Természetvédelmi Közlemények* 23: 100–117, .
20. Szép, T. and D. W. Gibbons (2000). "Monitoring of common breeding birds in Hungary using a randomised sampling design." *Ring* 22(2): 45-55.
21. Szép, T., K. Margóczi and A. Tóth (2011). "Biodiverzitás monitorozás [Biodiversity Monitoring], Nyíregyházi Főiskola " *Szegedi Tudományegyetem – Debreceni Egyetem, Nyíregyháza,* In Hungarian: 180.
22. Szép, T., K. Nagy, Z. Nagy and G. Halmo (2012). "*Population trends of common breeding and wintering birds in Hungary, decline of longdistance migrant and farmland birds during 1999–2012.*" Ornis Hungarica. Retrieved Published in. **20:** 13.
23. Takács, A. A., G. Takács and T. Lőrionc (2010). "*The Hungarian Nature Conservations Information System.*" Information about NCIS. Retrieved 22/11/17 from <http://www.termeszetvedelem.hu/_user/browser/File/TIR/TIR_eng.pdf> Published in English.
24. Takács, G. and Z. Molnár (2008). "Élőhely-térképezés [Monitoring Guide of Habitat Mapping], NBmR XI." *FHNPI – MTA ÖBKI, Sarród – Vácrátót,*: 69.
25. Török, K. and L. A. Fodor (2006). "*Nemzeti Biodiverzitás-monitorozó Rendszer - A Nemzeti Biodiverzitás-monitorozó Rendszer eredményei I. Élőhelyek, mohák és gombák. Környezetvédelmi és Vízügyi Minisztérium, Természetvédelmi Hivatal, Budapest. .*" National Biodiversity Monitoring System - Results of the National Biodiversity Monitoring System I. Habitats, mosses and fungi. Ministry of Environment and Water, Nature Conservation Office. Retrieved Published in Hungarian**:** pp. 197.
26. VM, P.-. (2013). "Útmutató a Natura 2000 fenntartási tervek készítéséhez [Guide to prepare Natura 2000 site management plans – in Hungarian]." *VM Természetmegőrzési Főosztály,*: 29.
27. Wolters Kluwer (1996). "*1996. évi LIII. törvény: a természet védelméről.*" ‘53th Law in 1996 about Nature Conservation’ (The Birds Directive (79/409) and Habitats Directive (92/43) were translated into this law) Retrieved 22/11/17 from <https://mkogy.jogtar.hu/?page=show&docid=99600053.TV#lbj0id9082> Published in Hungarian.

## Water Framework Directive

1. Ministry of Environment and Water Hungary (2007). "*WFD Article 8 Monitoring Programmes For Hungary.*" Hungarian Website for the Water Framework Directive. Retrieved 22/11/17 from <http://www.euvki.hu/docs/jel_2007/HU_RBD_Monitoring_HU1000.html> Published in English/ Hungarian.
2. Ministry of Environment and Water Hungary (2010). "*Vízgyűjtő-gazdálkodási Terv felülvizsgálata és a Kvassay Jenő Terv elkészítése tájékoztatás és társadalmi konzultáció.*" Review of the River Basin Management Plan and Preparation of the Kvassay Jeno Plan Information and Social Consultation Hungarian Website for the Water Framework Directive. Retrieved 22/11/17 from <http://www.euvki.hu/eng/index.html> Published in English/ Hungarian.
3. Országos Vízügyi Főigazgatóság, O. (2015). "*A projekt* " National Water Directorate. Retrieved 22/11/17 from <http://www.ovf.hu/hu/568313c3-eba5-441c-8c62-baf0244d2d00> Published in Hungarian.
4. Országos Vízügyi Főigazgatóság, O. (no date). "*General Directorate of Water Management.*" Retrieved 22/11/17 from <http://www.ovf.hu/en/> Published in English and Hungarian.
5. Országos Vízügyi Főigazgatóság, O. (no date). "*A Víz Keretirányelv előírásai szerinti monitoring vizsgálatok és az ahhoz szükséges fejlesztések végrehajtása.*" National Water Directorate - Implementation of monitoring studies and the necessary developments under the Water Framework Directive requirements. Retrieved 22/11/17 from <http://www.ovf.hu/hu/futo-projektek/617a5146-a88c-4c3c-85f7-77854497821d> Published in Hungarian.

# Romania

## Agri-environment schemes

1. Agenţia de Plăţi şi Intervenţie pentru Agricultură "*Anexa nr. 2 - Instrucţiuni De Completare A Formularului De Cerere Unică De Plată Informaţii Privind Schemele De Plată/Măsurile De Sprijin.*" Agency for Payments and Intervention for Agriculture - Instructions for Completing the Single Payment Application Form Payment Scheme Information / Support Measures. Retrieved 24/11/17 from <http://www.apia.org.ro/files/pages_files/Instructiuni_completare_cerere_unica_de_plata.pdf> Published in Romanian.
2. Agenţia de Plăţi şi Intervenţie pentru Agricultură "*Model Caiet de agro-mediu - Instrucțiuni De Completare A Caietelor De Agro-Mediu Și Agricultură Ecologică.*" Agro-Environment Book Model - Instructions for complementing the agro-environmental and containment areas ecological agriculture. Retrieved 24/11/17 from <http://www.apia.org.ro/files/pages_files/Anexa_nr._13_Caiet_de_agromediu_si_instructiuni_M214M10_P1236.pdf> Published in Romanian.
3. Agenţia de Plăţi şi Intervenţie pentru Agricultură (2017). "*Depune cererea unică de plată 2017 - vegetal şi zootehnie - Formular Cerere Unica 2017 / Legislaţie.*" Agency for Payments and Intervention for Agriculture - The single payment application 2017 - plant and animal husbandry - Single Application Form 2017 / Legislation. Retrieved 24/11/17 from <http://www.apia.org.ro/ro/formular-cerere-unica-2017-legislatie> Published in Romanian.
4. Agenţia de Plăţi şi Intervenţie pentru Agricultură (2017). "*Materiale informare - anul 2017.*" Agency for Payments and Intervention for Agriculture -
5. Information materials - 2017 - Concerning AES, modifications are included in general description on technical datasheet of the measures here. . Retrieved 24/11/17 from <http://www.apia.org.ro/ro/materiale-de-informare/materiale-informare1484830750> Published in Romanian.
6. Agenţia de Plăţi şi Intervenţie pentru Agricultură (2017). "*Materiale informare - anul 2017: Ghidul fermierului privind ecoconditionalitatea, .*" Farmer's Guide on Connectivity from the Agency for Payments and Intervention for Agriculture. Retrieved 15/11/17 from <http://www.apia.org.ro/ro/materiale-de-informare/materiale-informare1484830750> Published in Romanian.
7. Alcedo (2014). "*Ghid analize sol.*" Ground analysis guide - Alcedo offers soil analysis services. Retrieved 24/11/17 from <http://www.alcedoltd.ro/recomandari-tehnologice/ghid-analize-sol/> Published in Romanian.
8. Asociatia Producatorilor Si Importatorilor De Automobile (no date). Association of Manufacturers and Automobile Importers. Retrieved 24/11/17 from <http://www.anpm.ro/anpm_resources/migrated_content/uploads/16102_5%20SOL%202009.pdf> Published in Romanian.
9. Centrul National pentru Dezvoltare Durabila (2014). "*Ghid standard de monitorizare a speciilor de păsări de interes comunitar din România.*" National Center for Sustainable Development - Standard monitoring guide of bird species of Community interest from Romania. Retrieved 24/11/17 from <http://monitorizareapasarilor.cndd.ro/documents/Ghid-standard-de-monitorizare-pasari-2014.pdf> Published in Romanian.
10. Institutul Naţional De Cercetare-Dezvoltare Pentru Pedologie Agrochimie Şi Protecţia Mediului – Icpa Bucureşti (2011). "*Monitoringul Stării De Calitate A Solurilor Din - România [Soil Quality Monitoring In Romania]* ". Retrieved 24/11/17 from <http://www.unibuc.ro/prof/scradeanu_d/docs/2014/apr/16_19_20_25Monitoring_sol_ICPA_2011.pdf> Published in Romanian and English.
11. Lăcătuşu, R. (2007). "*Plan De Monitorizare A Amplasamentului Şi A Zonelor Adiacente Proiectului Roşia Montană Din Punctul De Vedere Al Impactului Asupra Solului Pentru Etapele De Construcţie, Exploatare, Închidere Şi Post- Închidere.*" Site Planning and Site Monitoring Plan Adjacent to the Roşia Montană Project from the Land Forward Impact Assessment Point for Construction, Exploitation, Closure and Post-Closure Stages. Retrieved 24/11/17 from <http://www.mmediu.ro/new/wp-content/uploads/Rosia_Montana/februarie_2011/Vol.-3_Rapoarte-si-Studii-Aditionale/Anexa_NE_Cap-4.4_01-Plan_monitorizare_Impact_Sol.pdf> Published in Romanian.
12. Meteo Romania (no date). "*CLIMA.*" Meteo Romania constantly analyze the climate fluctuations and develop projections of the evolution of the climate system both on a large scale and at the scale of Romania, transferring the scientific knowledge in the socio-economic environment through climatic products and services. Retrieved 24/11/17 from <http://www.meteoromania.ro/anm2/clima/> Published in Romanian.
13. Ministerul Agriculturii şi Dezvoltării Rurale (2016). "*Ordin privind aprobarea Programului național privind realizarea Sistemului național de monitorizare.*" Order on the approval of the National Program for the implementation of the National Monitoring System. Retrieved 24/11/17 from Monitorul Oficial Al României, Partea I, Nr. 928/28.XII.2011 at <http://old.madr.ro/pages/fond_funciar/ordin-278-din-9-decembrie-2011.pdf> Published in Romanian.
14. Ministerul Agriculturii și Dezvoltării Rurale (2016). "*Evaluarea on‐Going a PNDR  2014‐2020 în perioada 2017‐2020.*" On-Going benchmarking of NRDP 2014-2020 in the period 2017-2020. Retrieved 15/11/17 from <http://www.madr.ro/docs/dezvoltare-rurala/2017/Studiul-de-evaluare-I-RAI-2016.pdf> Published in Romanian.
15. Ministerul Agriculturii și Dezvoltării Rurale (no date). "*Ghid Informativ Pentru Beneficiarii Măsurilor De Mediu Și Climă Ale Programului Naţional De Dezvoltare Rurală (PNDR) 2014 - 2020.*" Information Guide for Beneficiaries of Environmental and Climate Measures Of the National Rural Development Program (PNDR) 2014-2020. Retrieved 24/11/17 from <http://www.apia.org.ro/files/pages_files/Ghid_informativ_pentru_beneficiarii_masurilor_de_mediu_si_clima_ale_Programului_National_de_Dezvoltare_Rurala_2014-2020.pdf> Published in Romanian.
16. Ministerul Agriculturii și Dezvoltării Rurale (no date). "*Lista datelor deschise la nivelul MADR.*" List of open data at MADR level. Retrieved 15/11/17 from <http://madr.ro/transparenta-institutionala/date-deschise/lista-datelor-deschise-la-nivelul-madr.html> Published in Romanian (and English).
17. Ministerul Agriculturii și Dezvoltării Rurale (no date). "*Proiecte depuse 2014 - 2020.*" Projects submitted 2014-2020. Retrieved 15/11/17 from <http://madr.ro/pndr-2014-2020/implementare-pndr-2014-2020/situatia-proiectelor-depuse-2014-2020.html> Published in Romanian (and English).
18. Ministerul Agriculturii și Dezvoltării Rurale (no date). "*Regulamentul de Organizare si Functionare al Comitetului de Monitorizare pentru Programul National de Dezvoltare Rurala 2014-2020.*" Organization and Functioning Regulation of the Monitoring Committee for the National Program for Rural Development 2014-2020. Retrieved 15/11/17 from <http://www.madr.ro/docs/dezvoltare-rurala/comitet-monitorizare/comitet_2017/ROF_CM__revizuit_23.03.2017.pdf> Published in Romanian.
19. Ministerul Agriculturii și Dezvoltării Rurale (no date). "*Reuniuni CM PNDR 2014 - 2020.*" Documents from the meetings of the NRDP 2014-2020. Retrieved 15/11/17 from <http://www.madr.ro/pndr-2014-2020/implementare-pndr-2014-2020/comitet-monitorizare/reuniuni-cm-pndr-2014-2020.html#page> Published in Romanian.
20. Ministerul Mediului (2017). "*Agenția Națională pentru Protecția Mediului.*" Ministry of the Environment National Agency for Environmental Protection - Main website. Retrieved 24/11/17 from <http://www.anpm.ro/> Published in Romanian.
21. Ministerul Mediului (2017). "*Agenția Națională pentru Protecția Mediului - 5. SOLUL.*" Ministry of the Environment National Agency for Environmental Protection - Soil information sheet. Retrieved 24/11/17 from <http://www.anpm.ro/anpm_resources/migrated_content/uploads/16102_5%20SOL%202009.pdf> Published in Romanian.
22. Ministerul Mediului (2017). "*Agenția Națională pentru Protecția Mediului - Sol-subsol.*" Ministry of the Environment National Agency for Environmental Protection - Soil and sub-soil. Retrieved 24/11/17 from <http://www.anpm.ro/sol-subsol> Published in Romanian.
23. Ministerul Mediului (2017). "*Atributiile Principale Pe Domeniul Substante Chimice Periculoase.*" Ministry of the Environment National Agency for Environmental Protection - Principal Dangers of Dangerous Chemicals. Retrieved 24/11/17 from <http://apmbn.anpm.ro/ro/substante-chimice-periculoase> Published in Romanian.
24. Programul Național de Dezvoltare Rurală (2017). "*Programul National de Dezvoltare Rurala 2014-2020 versiunea aprobata.*" National Rural Development Program 2014-2020 approved version. Retrieved 24/11/17 from <http://www.pndr.ro/implementare-pndr-2014-2020/pndr-2014-2020-versiune-aprobata.html> Published in Romanian.
25. Programul Național de Dezvoltare Rurală (no date). "*Raport anual PNDR 2014-2020.*" National Rural Development Program - Annual Report NRDP 2014-2020. Retrieved 24/11/17 from <http://www.pndr.ro/implementare-pndr-2014-2020/raport-anual-pndr-2014-2020.html> Published in Romanian.
26. Română, S. O. (no date). "*Custodii.*" Details of 32 protected natural areas. Retrieved 24/11/17 from <http://www.sor.ro/ro/mid/Custodii> Published in Romanian.
27. Romaniei, G. (2016). "*Controlul Prin Teledetecţie Addendum Naţional - 2016.*" Remote Sensing Control National Addendum - 2016. Retrieved 24/11/17 from <http://www.apia.org.ro/files/pages_files/Addendum_final_2016_(Modif).pdf> Published in Romanian.

## Natura 2000

1. European Environment Agency (2015). "*Eionet: European Topic Centre on Biological Diversity - State of Nature in the EU* " Reporting under the Birds and the Habitats Directives. Links give access to information on reporting under each directive. Retrieved 15/11/17 from <https://bd.eionet.europa.eu/activities/Reporting/Introduction> Published in English.
2. European Environment Agency (2017). "*Eionet - European Topic Centre on Biological Diversity: Reference Portal for Natura 2000.*" The Reference Portal for NATURA 2000 is part of the Standard Data Form (SDF). The portal provides those elements of the SDF which are subject to change over time and subject to changes due to technical developments. See paragraph 12) for the Threats and Pressures table. Retrieved 15/11/17 from <https://bd.eionet.europa.eu/activities/Natura_2000/reference_portal> Published in English.
3. Eurpoean Commission (no date). "*CIRCABC Communication and Information Resource Centre for Administrations, Businesses and Citizens.*" CIRCABC is an open-source, web-based application. It enables widespread collaborative groups to share information and resources in private workspaces over the web. Retrieved 15/11/17 from <https://circabc.europa.eu/faces/jsp/extension/wai/navigation/container.jsp> Published in English (options of multiple other languages).
4. Frederiksen, P., T. van der Sluis, A. Vadineanu, T. Terkenli, V. Gaube, A. Busck, J. P. Vesterager, N. Geamana, E. D. Schistou and B. Pedroli (2017). "Misfits and compliance patterns in the transposition and implementation of the Habitats Directive—four cases. ." *Land Use Policy*(62): 337-350. <http://dx.doi.org/10.1016/j.landusepol.2016.12.010>.
5. IBIS (2007). "*Registrul National Integrat.*" National Integrated Registry. Retrieved 15/11/17 from <http://ibis.anpm.ro/Default.aspx> Published in Romanian.
6. Milvus Group (2017). "*Milvus Group* " Website detailing the work of Milvus Group, who have 11 working groups for the protection of nature. . Retrieved 15/11/17 from <http://milvus.ro/RO/> Published in Romanian, English or Hungarian.
7. Ministerul Mediului (2017). "*Planuri de management aprobate.*" Excel spreadsheet of approved management plans produced by the Ministry of Environment. Retrieved 15/11/17 from <http://www.mmediu.gov.ro/app/webroot/uploads/files/Planuri_de_management_aprobate_si_numar_MO_27.02.2017.xlsx> Published in Romanian.
8. Ministerul Mediului: Agenția Națională pentru Protecția Mediului (2014). "*Ministerial Order no. 1052/2014 Privind aprobarea Metodologiei de atribuire în administrare și custodie a ariilor naturale protejate.*" Regarding the approval of the methodology for awarding administration and custody of protected natural areas; Its Annex 5 provides the Structure of the annual activity report of administrators / custodians of protected natural areas and according to this structure, the Anthropogenic and natural pressures exerted on the protected natural area must be reported. Retrieved 15/11/17 from <http://www.anpm.ro/documents/12220/2552946/Ord.+1052-2014.pdf/f8a98925-c2a6-441c-aad4-42bc312bcb98> Published in Romanian.
9. Societatea Ornitologică Română (no date). "*Ce facem.*" The work and mission of the Bird Society of Romania. . Retrieved 15/11/17 from <http://sor.ro/ro/mid/Despre-Noi/Ce-facem-misiune-obiective.html> Published in Romanian.

## Water Framework Directive

1. Administraţia Naţională Apele Române (2016). "*Planul de Management Bazinal.*" Basin Management Plan for the Danube Delta, with annexes. Retrieved 15/11/17 from <http://www.rowater.ro/dadobrogea/Planul%20de%20Management%20Bazinal/Forms/AllItems.aspx?RootFolder=%2fdadobrogea%2fPlanul%20de%20Management%20Bazinal%2fPlan%20de%20Management%20actualizat%20al%20Fluviului%20Dunarea%2c%20Deltei%20Dunarii%2c%20SH%20Dobrogea%20si%20Apelor%20Costiere%202016-2021&FolderCTID=&View=%7b41309FF5-1279-4CB7-BD7F-07006BCC401F%7d> Published in Romanian.
2. Administraţia Naţională Apele Române (2017) "*Legislation*." Links to specific legislation relating to the “Romanian Waters” National Administration. Retrieved 14/11/17 from <http://www.rowater.ro/LegislatieOrganizarea%20si%20functionarea%20institutiei/Legislatie.aspx> Published in Romanian.
3. Administraţia Naţională Apele Române (no date). "*Anexele Planului Național de Management actualizat.*" Annexes to the updated National Management Plan. Retrieved 14/11/17 from <http://www.rowater.ro/TEST/Planul%20National%20de%20Management%202010%20-%20Sinteza%20planurilor%20de%20management%20la%20nivel%20de%20bazine-spatii%20hidrografice/Anexe%201-6%20PlanNationalManagement%20-%20vol.II.pdf> Published in Romanian.
4. Administraţia Naţională Apele Române (no date). "*Planul Naţional De Management Actualizat Aferent Porţiunii Din Bazinul Hidrografic Internaţional Al Fluviului Dunărea* " Updated National Management Plan for the Danube River Basin. Retrieved 14/11/17 from <http://www.rowater.ro/TEST/Planul%20Na%C8%9B.%20de%20Manag%20actualizat%202016-2021-Sinteza%20Planurilor%20de%20Manag.%20la%20nivel%20de%20bazine-spa%C8%9Bii%20hidrografice%20actualizate/Planul%20National%20de%20Management%20actualizat.pdf> Published in Romanian.
5. Administraţia Naţională Apele Române (no date). "*Proiect de Declarație privind Evaluarea Strategică de Mediu pentru Planul național de management actualizat aferent porțiunii din bazinul hidrografic internațional al fluviului Dunărea care este cuprinsă în teritoriul României.*" Draft Statement on Strategic Environmental Assessment for the updated National Management Plan for the portion of the Danube international river basin included in the territory of Romania. Retrieved 14/11/17 from <http://www.rowater.ro/TEST/Declara%C8%9Bie%20SEA%20pentru%20Planul%20Na%C8%9Bional%20de%20Management%20actualizat/Declaratie%20SEA%20pentru%20Planul%20Na%C8%9Bional%20de%20Management%20actualizat.pdf> Published in Romanian.
6. Rîşnoveanu, G., G. Chiriac and M. Moldoveanu (2017). "Robustness of the biotic indicators used for classification of ecological status of lotic water bodies: a testing method when the data series are short." *Ecological indicators* 76: 170-177. <http://dx.doi.org/10.1016/j.ecolind.2016.11.044>.

# Scotland (UK)

## Agri-environment schemes

1. Bidoversity Scotland (2017). "*The 2020 Challenge for Scotland's Biodiversity.*" Retrieved 23/11/17 from <http://www.biodiversityscotland.gov.uk/doing/strategy/> Published in English.
2. European Commission (2015). "*Annual implementation report United Kingdom - Rural Development Programme (Regional) - Scotland.*" Retrieved 23/11/17 from <http://www.gov.scot/Resource/0050/00501356.pdf> Published in English.
3. European Commission (2016). "*United Kingdom - Rural Development Programme (Regional) - Scotland.*" Retrieved 23/11/17 from <http://www.gov.scot/Resource/0050/00501661.pdf> Published in English.
4. Scottish Government (2009). "*Monitoring & Evaluation of Agrienvironment Schemes* " Final Report, May 2009 Retrieved 23/11/17 from <http://www.gov.scot/Resource/Doc/289188/0088491.pdf> Published in English.
5. Scottish Government (2013). "*Scotland Rural Development Programme (SRDP) 2014-2020 Stage 1 Consultation.*" Search for “Focus areas under each RDP priority” for an overview. Retrieved from <http://www.gov.scot/Publications/2013/05/9633/19> Published in English.
6. Scottish Government (2014). "*Ex-Ante Evaluation of Scotland’s Rural Development Programme 2014-2020.*" Written by Agra CEAS Consulting. Retrieved 23/11/17 from <http://www.gov.scot/Resource/0047/00475153.pdf> Published in English.
7. Scottish Government (2014). "*Scotland Rural Development Programme (SRDP) 2014-2020 Stage 2: Final Proposals.*" Section 9: Agri-Environment-Climate Scheme. Retrieved 23/11/17 from <http://www.gov.scot/Publications/2013/12/7550/291107> Published in English.
8. Scottish Government (2014). "*Scotland Rural Development Programme (SRDP) 2014-2020 Stage 2: Final Proposals.*" Executive Summary. Retrieved 23/11/17 from <http://www.gov.scot/Publications/2013/12/7550/291098> Published in English.
9. Scottish Government (2015). "*2015 No. 192: Agriculture: The Rural Development (Scotland) Regulations 2015* ". Retrieved 23/11/17 from <http://extwprlegs1.fao.org/docs/pdf/uk145117.pdf> Published in English.
10. Scottish Government (2015). "*AECS targeting and budget allocations per option/region.*" Part of the Scottish Rural Development Programme. Retrieved 23/11/17 from <http://www.gov.scot/Resource/0047/00476444.pdf> Published in English.
11. Scottish Government (2015). "*Ex-Ante Evaluation, Strategic Environmental Assessment and Business and Regulatory Impact Assessment (BRIA).*" Retrieved 23/11/17 from <http://www.gov.scot/Topics/farmingrural/SRDP/SRDP20142012/SRDP20142020ExAnteEvaluationSEA> Published in English.
12. Scottish Government (2015). "*Measuring The Natural Heritage Outcomes Resulting From The Biodiversity Measures In The 2007-2013 Scotland Rural Development Programme: Monitoring Results Part 1-4.*" Retrieved 23/11/17 from <http://www.gov.scot/Resource/0049/00492399.pdf> Published in English.
13. Scottish Government (2015). "*Scottish Rural Development Programme:Summary of Progress in 2014 & 2015.*" Retrieved 23/11/17 from <http://www.gov.scot/Resource/0051/00514108.pdf> Published in English.
14. Scottish Government (2016). "*Monitoring the environmental outcomes delivered by the 2014-20 SRDP Agri-Environment Climate Scheme.*" The aim of this research project is to measure the environmental benefits resulting from management under the 2014-20 SRDP Agri-Environment Climate Scheme. Retrieved 23/11/17 from <https://www.publiccontractsscotland.gov.uk/search/show/search_view.aspx?ID=MAR238898> Published in English.
15. Scottish Government (2016). "*Rural Payments and Services: Agri-Environment Climate Scheme - Endorsements.*" Retrieved 23/11/17 from <https://www.ruralpayments.org/publicsite/futures/topics/all-schemes/agri-environment-climate-scheme/agri-environment-climate-scheme-full-guidance-menu/agri-environment-endorsements/> Published in English.
16. Scottish Government (2017). "*Ex-Post Evaluation of the Scotland Rural Development Programme 2007-2013.*" Page 5. Retrieved 23/11/17 from <http://www.gov.scot/Resource/0051/00515117.pdf> Published in English.
17. Scottish Government (2017). "*National Performance Framework.*" The National Performance Framework provides a clear vision for Scotland with broad measures of national wellbeing covering a range of economic, health, social and environmental indicators and targets. Retrieved 23/11/17 from <http://www.gov.scot/About/Performance/purposestratobjs> Published in English.
18. Scottish Government (2017). "*Rural Payments and Services: Agri-Environment Climate Scheme.*" Retrieved 23/11/17 from <https://www.ruralpayments.org/publicsite/futures/topics/all-schemes/agri-environment-climate-scheme/> Published in English.
19. Scottish Government (2017). "*Scottish Rural Development Programme 2014 - 2020 Monitoring and Evaluation.*" Retrieved 23/11/17 from <http://www.gov.scot/Topics/farmingrural/SRDP/SRDP2014-2020MonitoringandEvaluation> Published in English.
20. Scottish Government (2017). "*Slurry Storage.*" Guidance on sufficient slurry storage. Retrieved 23/11/17 from <https://www.ruralpayments.org/publicsite/futures/topics/all-schemes/agri-environment-climate-scheme/management-options-and-capital-items/slurry-storage> Published in English.
21. Scottish Government (2017). "*SRDP 2014 - 2020 Monitoring and Evaluation Steering Group.*" Retrieved 23/11/17 from <http://www.gov.scot/Topics/farmingrural/SRDP/SRDP2014-2020MonitoringandEvaluation/SG> Published in English.
22. Scottish Government (2017). "*SRDP 2014 - 2020 Rural Development Operational Committee (RDOC).*" Retrieved 23/11/17 from <http://www.gov.scot/Topics/farmingrural/SRDP/SRDP2014-2020RDOC> Published in English.
23. Scottish Government (no date). "*Inspections checker.*" Rural Payments and Services - RPID inspections are checking the performance of farmers’ implementation of EU money. In case of AECS there are Capital inspection, Land inspection and Cross Compliance. Retrieved 23/11/17 from <https://www.ruralpayments.org/publicsite/futures/topics/inspections/inspections-checker/> Published in English.
24. Scottish Natural Heritage (SNH) (2017). "*Beetle Banks: Updated map for 2016.*" Retrieved 23/11/17 from <https://www.ruralpayments.org/publicsite-rest/fscontent/repository/portal-system/mediadata/media/resources/updated_targeting_map_-_beetlebanks_16_12_2015.pdf?sourcePage=https://www.ruralpayments.org/publicsite/futures/topics/updates/guidance-archive/agri-environment-climate-scheme-management-options-archive/beetlebanks-archived-18-01-2017> Published in English.

## Natura 2000

1. British Trust for Ornithology (no date). "*The Wetland Bird Survey (WeBS).*" WeBS homepage. The Wetland Bird Survey (WeBS) monitors non-breeding waterbirds in the UK. . Retrieved 23/11/17 from <https://www.bto.org/volunteer-surveys/webs> Published in English.
2. Joint Nature Conservation Committee (JNCC) (2004). "*Common Standards MonitoringIntroduction to the Guidance Manual* " Monitoring guidance has been produced by the Joint Nature Conservation Committee, and is called Common Standard Monitoring Guidance Manual. Retrieved 23/11/17 from <http://jncc.defra.gov.uk/pdf/CSM_introduction.pdf> Published in English.
3. Joint Nature Conservation Committee (JNCC) (2007). "*The Conservation of Habitats and Species Regulations 2010.*" Retrieved 23/11/17 from <http://jncc.defra.gov.uk/page-1379> Published in English.
4. Joint Nature Conservation Committee (JNCC) (2007). "*The Offshore Marine Conservation (Natural Habitats, & c.) Regulations 2007.*" Retrieved 23/11/17 from <http://jncc.defra.gov.uk/page-4550> Published in English.
5. Joint Nature Conservation Committee (JNCC) (2010). "*Common Standards Monitoring for Designated Sites: First Six Year Report.*" Retrieved 23/11/17 from <http://jncc.defra.gov.uk/page-3520> Published in English.
6. Joint Nature Conservation Committee (JNCC) (2010). "*Special Areas of Conservation (SAC).*" Retrieved 23/11/17 from <http://jncc.defra.gov.uk/page-23> Published in English.
7. Joint Nature Conservation Committee (JNCC) (2014). "*Effective use of opportunistic data.*" Retrieved 23/11/17 from <http://jncc.defra.gov.uk/page-6858> Published in English.
8. Joint Nature Conservation Committee (JNCC) (2014). "*EU Habitats Directive Reporting.*" Every six years, Member States of the European Union are required to report on implementation of the Habitats Directive, under Article 17 of the Directive. Retrieved 23/11/17 from <http://jncc.defra.gov.uk/default.aspx?page=6397> Published in English.
9. Joint Nature Conservation Committee (JNCC) (2014). "*Genetically Modified (GM) crops and biodiversity.*" Retrieved 23/11/17 from <http://jncc.defra.gov.uk/page-6869> Published in English.
10. Joint Nature Conservation Committee (JNCC) (2014). "*Impacts of Nitrogen Deposition on Vegetation.*" Retrieved 23/11/17 from <http://jncc.defra.gov.uk/page-6871> Published in English.
11. Joint Nature Conservation Committee (JNCC) (2014). "*Seabird 2000.*" Seabird 2000 was the third complete census of the entire breeding seabird population of Britain and Ireland. Results found here. . Retrieved 23/11/17 from <http://jncc.defra.gov.uk/page-1548> Published in English.
12. Scottish National Heritage (SNH) (2017). "*European protected species.*" Certain species listed on Annex IV of the Habitats Directive are given special protection in Scotland as European protected species. Retrieved 23/11/17 from <https://www.snh.scot/professional-advice/safeguarding-protected-areas-and-species/protected-species/legal-framework/habitats-directive-and-habitats-regulations/european> Published in English
13. Scottish National Heritage (SNH) (2017). "*How we monitor features.*" Scottish Natural Heritage, volunteers and specialist contractors carry out site condition monitoring fieldwork to assess natural features against UK-wide standards. Retrieved 23/11/17 from <https://www.snh.scot/professional-advice/safeguarding-protected-areas-and-species/protected-areas/site-condition-monitoring/how-we-monitor-features> Published in English
14. Scottish National Heritage (SNH) (2017). "*Natura sites.*" Natura sites – Special Areas of Conservation and Special Protection Areas – are designated under the European Habitats and Birds Directives. Retrieved 23/11/17 from <https://www.snh.scot/professional-advice/safeguarding-protected-areas-and-species/protected-areas/international-designations/natura-sites> Published in English
15. Scottish National Heritage (SNH) (2017). "*Pressures on feature condition.*" As part of the site condition monitoring fieldwork, SNH inspect sites for factors that may negatively or positively affect features. Retrieved 23/11/17 from <https://www.snh.scot/professional-advice/safeguarding-protected-areas-and-species/protected-areas/site-condition-monitoring/pressures-feature-condition> Published in English
16. Scottish National Heritage (SNH) (2017). "*Site condition monitoring.*" The Site Condition Monitoring programme assesses the condition of natural features of special interest on designated sites in Scotland. Retrieved 23/11/17 from <https://www.snh.scot/professional-advice/safeguarding-protected-areas-and-species/protected-areas/site-condition-monitoring> Published in English
17. Scottish National Heritage (SNH) (2017). "*Use of monitoring results.*" Scottish Natural Heritage and partner government agencies use the results of Site Condition Monitoring programme in various ways. Retrieved 23/11/17 from <https://www.snh.scot/professional-advice/safeguarding-protected-areas-and-species/protected-areas/site-condition-monitoring/use-monitoring-results> Published in English
18. Scottish National Heritage (SNH) (no date). " *Sitelink. S*iteLink provides easy access to data and information on key Protected Areas across Scotland ranging from sites of local natural heritage to designations of national and international importance.” Retrieved 23/11/17 from <http://gateway.snh.gov.uk/sitelink/index.jsp> Published in English
19. The UK National Archives (1994). "*The Conservation (Natural Habitats, &c.) Regulations 1994.*" Retrieved 23/11/17 from <http://www.legislation.gov.uk/uksi/1994/2716/contents/made> Published in English.
20. Williams, J. M. (2006). "Common Standards Monitoring for Designated Sites: First Six Year Report. Peterborough." *JNCC*. Retrieved 23/11/17 from <http://jncc.defra.gov.uk/page-3520> Published in English.

## Water Framework Directive

1. Critchlow-Watton, N., K. Davidson, I. Malcolm, I. Sime and C. Sinclair (2013). "*Freshwater Monitoring Action Plan.*" Retrieved 23/11/17 from <https://www.environment.gov.scot/media/1526/scotlands-freshwater-monitoring-action-plan-april-2014.pdf> Published in English.
2. Environment Agency (2014). "*Update to the draft river basin management plans.*" Retrieved 23/11/17 from <https://www.gov.uk/government/consultations/update-to-the-draft-river-basin-management-plans> Published in English.
3. Scotland's Environment (no date). "*Citizen Science Portal.*" A place to share data and ideas. Retrieved 23/11/17 from <https://envscot-csportal.org.uk/> Published in English.
4. Scotland's Environment (no date). "*Get involved.*" Examples of citizen science. Retrieved 23/11/17 from <https://www.environment.gov.scot/get-involved/> Published in English.
5. Scottish Environment Protection Agency (SEPA) (2005). "*Introduction to the Controlled Activities Regulations.*" This paper is intended to provide a simple explanation of the Controlled Activities Regulations (Regulations) 2005 (CAR). Retrieved 23/11/17 from <https://www.sepa.org.uk/media/34800/introduction-to-the-controlled-activities-regulations.pdf> Published in English.
6. Scottish Environment Protection Agency (SEPA) (2007). "*Scotland’s WFD aquatic monitoring strategy* " First released version of WFD aquatic monitoring strategy. Retrieved 23/11/17 from <https://www.sepa.org.uk/media/38220/wfd_aquatic_monitoring_strategy-scotland_river_basin.pdf> Published in English.
7. Scottish Environment Protection Agency (SEPA) (2008). "*Solway-Tweed WFD aquatic monitoring strategy* ". Retrieved 23/11/17 from <https://www.sepa.org.uk/media/38207/solway_tweed_wfd_aquatic_monitoring_strategy.pdf> Published in English.
8. Scottish Environment Protection Agency (SEPA) (2015). "*State of Scotland’s Water Environment 2015 Summary Report.*" Retrieved 23/11/17 from <https://www.sepa.org.uk/media/286883/state-of-scotland-s-water-environment-2015-summary-report.pdf> Published in English.
9. Scottish Environment Protection Agency (SEPA) (2017). "*River Basin Management Planning.*" Information on RBMP from SEPA's website. Retrieved 23/11/17 from <https://www.sepa.org.uk/environment/water/river-basin-management-planning/> Published in English.
10. Scottish Environment Protection Agency (SEPA) (2017). "*Supporting Guidance (WAT-SG-67)Assessing the Significance of Impacts - Social, Economic and Environmental.*" Retrieved 23/11/17 from <https://www.sepa.org.uk/media/149801/wat_sg_67.pdf> Published in English.
11. Scottish Environment Protection Agency (SEPA) (no date). "*Actions to deliver RBMP.*" Retrieved 23/11/17 from <https://www.sepa.org.uk/environment/water/river-basin-management-planning/actions-to-deliver-rbmp/> Published in English.
12. Scottish Environment Protection Agency (SEPA) (no date). "*Information Sheet: Pardovan Burn* " Example of an individual water body annual classification sheet from The Water Environment Hub <https://www.sepa.org.uk/data-visualisation/water-environment-hub/>. Retrieved 23/11/17 from <https://www.sepa.org.uk/data-visualisation/water-environment-hub/?display=information_sheet&waterbodyid=3400> Published in English.
13. Scottish Environment Protection Agency (SEPA) (no date). "*Monitoring.*" Water Monitoring Information. Retrieved 23/11/17 from <https://www.sepa.org.uk/environment/water/monitoring/> Published in English.
14. Scottish Environment Protection Agency (SEPA) (no date). "*RBMP information sources.*" Retrieved 23/11/17 from <https://www.sepa.org.uk/environment/water/river-basin-management-planning/rbmp-information-sources/> Published in English.
15. Scottish Environment Protection Agency (SEPA) (no date). "*Water.*" Explains the regulations that protect Scotland’s water environment. Retrieved 23/11/17 from <https://www.sepa.org.uk/regulations/water/> Published in English.
16. Scottish Environment Protection Agency (SEPA) (no date). "*Water Bodies Data Sheets.*" Retrieved 23/11/17 from <http://apps.sepa.org.uk/waterbodydatasheets/> Published in English.
17. Scottish Environment Protection Agency (SEPA) (**no date**). "*Who is involved with RBMP.*" Retrieved 23/11/17 from <https://www.sepa.org.uk/environment/water/river-basin-management-planning/who-is-involved-with-rbmp/> Published in English.
18. Scottish Environmental Protection Agency (SEPA) (2011). "*The Water Environment (Controlled Activities) (Scotland) Regulations 2011 (as amended).*" A practical guide to the regulations, including: An overview, definitions of the regimes, levels of authorisation, the General Binding Rules. Retrieved 23/11/17 from <https://www.sepa.org.uk/media/34761/car_a_practical_guide.pdf> Published in English.
19. Scottish Environmental Protection Agency (SEPA) (2013). "*The Water Environment (Controlled Activities) (Scotland) Amendment Regulations 2013.*" Retrieved 23/11/17 from <http://www.legislation.gov.uk/ssi/2013/176/policy-note/contents> Published in English.
20. Scottish Government (2011). "*Scottish Environmental Monitoring Strategy.*" Retrieved 23/11/17 from <http://www.gov.scot/Resource/Doc/364186/0123758.pdf> Published in English.
21. Scottish Government (2014). "*Environmental Protection: The Scotland River Basin District (Standards) Directions 2014* ". Retrieved 23/11/17 from <http://www.gov.scot/Resource/0045/00457867.pdf> Published in English.
22. Scottish Government (2015). "*The river basin management plan for the Scotland river basin district: 2015–2027.*" Retrieved 23/11/17 from <https://www.sepa.org.uk/media/163445/the-river-basin-management-plan-for-the-scotland-river-basin-district-2015-2027.pdf> Published in English.
23. Scottish Government (2016). "*River Basin Management Planning Framework.*" Retrieved 23/11/17 from <http://www.gov.scot/Topics/Environment/Water/15561/WFD/RBMPframework> Published in English.
24. Scottish Natural Heritage (SNH) (2017). "*Safeguarding protected areas and species.*" Retrieved 23/11/17 from <https://www.snh.scot/professional-advice/safeguarding-protected-areas-and-species> Published in English.
25. Scottish Natural Heritage (SNH) (2017). "*Water Framework Directive.*" Retrieved 23/11/17 from <https://www.snh.scot/professional-advice/safeguarding-protected-areas-and-species/safeguards-beyond-protected-areas/water-framework-directive> Published in English.
26. The UK National Archives (2011). "*The Water Environment (Controlled Activities) (Scotland) Regulations 2011.*" Retrieved 23/11/17 from <http://www.legislation.gov.uk/ssi/2011/209/contents/made> Published in English.
27. The UK National Archives (2013). "*The Water Environment (Controlled Activities) (Scotland) Amendment Regulations 2013.*" Retrieved 23/11/17 from <http://www.legislation.gov.uk/ssi/2013/176/contents/made> Published in English.
28. Water Framework Directive – United Kingdom Technical Advisory Group(WFD-UKTAG) (2014). "*UKTAG Lake Assessment Method Phytoplankton: Phytoplankton Lake Assessment Tool with Uncertainty Module (PLUTO).*" Retrieved 20/11/17 from <http://wfduk.org/sites/default/files/Media/Characterisation%20of%20the%20water%20environment/Biological%20Method%20Statements/Lake%20Phytoplankton%20UKTAG%20Method%20Statement.pdf> Published in English.
29. Water Framework Directive – United Kingdom Technical Advisory Group(WFD-UKTAG) (2015). "*UKTAG River Assessment Method River continuity: Barrier to fish migration method (Scotland).*" Retrieved 20/11/17 from <https://www.wfduk.org/sites/default/files/Media/Characterisation%20of%20the%20water%20environment/Biological%20Method%20Statements/Barrier%20to%20fish%20migration%20%28Scotland%29%20Method%20Statement.pdf> Published in English.
30. Water Framework Directive United Kingdom Technical Advisory Group(WFD-UKTAG) (2014). "*UKTAG work priorities 2014-2017.*" Retrieved 20/11/17 from <https://www.wfduk.org/sites/default/files/Media/UKTAG%20high%20level%20work%20priorities%202014-2017%2020140312%20v2.pdf> Published in English.

# Slovakia

## Agri-environment schemes

1. European Commission (2017). "*Agriculture and Rural - Country Files: Slovakia.*" Retrieved 28/11/17 from <https://ec.europa.eu/agriculture/rural-development-2014-2020/country-files/sk_en> Published in English.
2. Národné poľnohospodárske a potravinárske centrum (no date). "*Záverečné a hodnotiace správy k úlohe technickej pomoci: Priebežné (ongoing) hodnotenie Programu rozvoja vidieka SR 2007 - 2013.*" National Agricultural and Food Center - Final and evaluation reports on the role of technical assistance: Continuous evaluation of the rural development program 2007-2013. Retrieved 24/11/17 from <http://www.vuepp.sk/04_ongoing.htm> Published in Slovak (site also in English).
3. PPA - Pôdohospodárska platobná agentúra (2017). "*Agricultural Paying Agency (APA).*" Retrieved 28/11/17 from <http://www.apa.sk/en/> Published in English and Slovakian.

## Natura 2000

1. Janák, M., J. Černecký and A. Saxa (2015). "*Monitoring of animal species of Community interest in the Slovak Republic, results and assessment in the period of 2013 – 2015.*" Banská Bystrica: State Nature Conservancy of the Slovak Republic: 300 pp. ISBN 978-380-8184-8022-8187. Published in English. Retrieved 28/11/17 from <http://daphne.sk/wp-content/uploads/2013/12/monitoring_zooANGL_web.pdf> Published in English.
2. Šefferová Stanová, V., J. Galvánková and I. Rizman (2015). *"Monitoring of plants and habitats of European importance in Slovak republic. Results and assessment for years 2013 – 2015.*" Banská Bystrica: The State Nature Conservancy of Slovak Republic /Štátna ochrana prírody Slovenskej republiky*.* Retrieved 28/11/17 from <http://daphne.sk/wp-content/uploads/2013/12/monitoring_botANGL_web.pdf> Published in English.
3. SOS/BirdLife Slovensko (2013). "*Methods of systematic long-term monitoring of selective bird species in Protected Bird Areas.”* The State Nature Conservancy of Slovak Republic/Štátna ochrana prírody Slovenskej republiky*.* Banská Bystrica. Pp. 184. Published in Slovakian.
4. Štátna ochrana prírody slovenskej republiky (no date). "*Komplexný informačný monitorovací systém.*" State Nature Conservation of the Slovak Republic - Comprehensive information monitoring system. Retrieved 24/11/17 from <http://www.biomonitoring.sk/> Published in Slovakian.

## Water Framework Directive

1. Ministerstvo Životného Prostredia Slovenskej Republiky (2015). "*Vodný Plán Slovenska - Plán manažmentu správneho územia povodia Dunaja: Aktualizácia.*" Ministry of the Environment of the Slovak Republic - Water Plan of Slovakia - Management plan of the river basin district Danube: update. Retrieved 28/11/17 from <https://www.minzp.sk/files/sekcia-vod/vodny-plan-2015/vodny-planslovenska-2015_sup-dunaja-sup-visly.pdf> Published in Slovakian.
2. Slovenská agentúra životného prostredia (2015). "*Ochrana Vôd.*" Protection of the Overseas - Web-portal where monitoring data is updated regularly. Retrieved 28/11/17 from <http://old.sazp.sk/public/index/go.php?id=1167&lang=sk> Published in Slovakian.
3. Slovenskŷ Hydrometeorologickŷ ûstav (2015). "*Celkové hodnotenie kvality podzemných vôd na Slovensku v roku 2015.*" Slovak Hydrometeorological Institute - Overall assessment of groundwater quality in Slovakia in 2015. Retrieved 28/11/17 from <http://www.shmu.sk/File/podzemna%20voda/Kvalita/Text/2015/verejne_informacie_2015.pdf> Published in Slovakian.
4. Slovenskŷ Hydrometeorologickŷ ûstav (2017). "*Periodicky vydávané publikácie.*" Slovak Hydrometeorological Institute - Publications. Retrieved 28/11/17 from <http://www.shmu.sk/sk/?page=1597> Published in Slovakian.
5. Výskumný ústav vodného hospodárstva (2017). "*Plány manažmentu čiastkových povodí.*" Water Research Institute - River Basin Management Plans. Retrieved 28/11/17 from <http://www.vuvh.sk/rsv2/default.aspx?pn=PMCP2> Published in Slovakian.
6. Výskumný ústav vodného hospodárstva (2017). "*Úvod.*" Water Research Institute - Introduction. Retrieved 28/11/17 from <http://www.vuvh.sk/rsv2/> Published in Slovakian.
7. Výskumný ústav vodného hospodárstva (no date). "*Podzemné vody - Kvalita - Monitorovacia sieť a rozsah sledovaných ukazovateľov.*" Water Research Institute - Groundwater - Quality - Monitoring network and range of indicators. Retrieved 28/11/17 from <http://www.vuvh.sk/RSV2/download/02_Dokumenty/26_Ramcovy_program_monitorovania_vod/P4_2_3.pdf> Published in Slovakian.
8. Výskumný ústav vodného hospodárstva (no date). "*Podzemné vody - Kvantita - Monitorovacia sieť a rozsah sledovaných parametrov.*" Water Research Institute - Groundwater - Quantity - Monitoring network and scope of monitored parameters. Retrieved 28/11/17 from <http://www.vuvh.sk/RSV2/download/02_Dokumenty/26_Ramcovy_program_monitorovania_vod/P4_2_2.pdf> Published in Slovakian.
9. Výskumný ústav vodného hospodárstva (no date). "*Zoznam vodomerných staníc štátnej pozorovacej siete SR.*" Water Research Institute - Surface water quantity: List of water stations of the national observation network SR (417 + 6 new). Retrieved 28/11/17 from <http://www.vuvh.sk/rsv2/default.aspx?pn=PMCP2> Published in Slovakian.
10. Za živé rieky (2017). "*Ochrana vôd v SR a EÚ.*" For Lively Rivers - Water protection in Slovakia and the EU. Retrieved 28/11/17 from <http://ziverieky.sk/ochrana-vod-na-slovensku/> Published in Slovakian.

# Sweden

## Agri-environment schemes

1. Bergström, S. (2014). "*Transport of per- and polyfluoroalkyl substances in soil and groundwater in Uppsala, Sweden.*" Retrieved 28/11/17 from <https://stud.epsilon.slu.se/7392/18/bergstrom_s_141008.pdf> Published in English
2. European Commission (2017). "*Sweden: CAP in Your Country.*" Retrieved 28/11/17 from <https://ec.europa.eu/agriculture/sites/agriculture/files/cap-in-your-country/pdf/se_en.pdf> Published in English.
3. Hedlund, J. (2015). "*Per- and polyfluoroalkyl substances (PFASs) in Swedish waters.*" Retrieved 28/11/17 from <https://stud.epsilon.slu.se/9187/7/hedlund_j_160610.pdf> Published in English
4. Jordbruks verket (2015). "*Jordbruksverkets genomförandeplan för att nå miljömålen.*" The Swedish Agricultural Agency's Agricultural Agency's implementation plan for achieving environmental goals. Retrieved 28/11/17 from <http://www.jordbruksverket.se/download/18.3734b3c1155b9c6933bd372a/1467795217570/Jordbruksverkets+genomf%C3%B6randeplan.pdf> Published in Sweden.
5. Naturvårdsverket (2008). "*Jordbruksmark - revision av nationell miljöövervakning.*" Environmental Protection Agency - Land: audit of national environmental monitoring. Retrieved 28/11/17 from <http://www.naturvardsverket.se/Documents/publikationer/620-5824-1.pdf?pid=3427>) Published in Swedish.
6. Naturvårdsverket (2011). "*Miljöeffekter av EU:s - jordbrukspolitik.*" Environmental Protection Agency - Environmental impacts of EU agricultural policy. Retrieved 28/11/17 from <http://www.naturvardsverket.se/Documents/publikationer6400/978-91-620-6461-7.pdf?pid=3769> Published in Swedish.
7. Naturvårdsverket (2017). "*Åtgärdsprogram för bevarande av hotade arter.*" Environmental Protection Agency - Action program for conservation of threatened species. Retrieved 28/11/17 from <http://www.naturvardsverket.se/Global-meny/Sok/?query=%C3%85tg%C3%A4rdsprogram+f%C3%B6r+bevarande+av+hotade+arter&Naturvardsverketfv=6&sort=date> Published in Swedish.
8. Naturvårdsverket (2017). "*Åtgärdsprogram för hotade arter och naturtyper.*" Environmental Protection Agency - Action program for threatened species and habitats. Retrieved 28/11/17 from <http://www.naturvardsverket.se/Miljoarbete-i-samhallet/Miljoarbete-i-Sverige/Uppdelat-efter-omrade/Naturvard/Atgardsprogram-for-hotade-arter/> Published in Swedish.
9. SLU Sveriges lantbruksuniversitet (Swedish University of Agricultural Sciences) (2017). "*Nationell inventering av landskapet i Sverige, NILS.*" National inventory of the landscape in Sweden, NILS. Retrieved 28/11/17 from <https://www.slu.se/centrumbildningar-och-projekt/nils/> Published in English (site also in Swedish).
10. SLU Sveriges lantbruksuniversitet (Swedish University of Agricultural Sciences) (2017). "*Uppföljning av förändringar i jordbrukslandskapet.*" Follow-up of changes in the agricultural landscape. Retrieved 28/11/17 from <https://www.slu.se/centrumbildningar-och-projekt/nils/uppfoljning-av-kvalitetsforandringar-i-angs-och-betesmarker/> Published in English (site also in Swedish).
11. Svenska Dagbladet "*Livsmedelsverket: gift i dricksvattet troligt i fler kommuner.*" The Food Administration: Probably in the drinking water, poison in several municipalities (news report). Retrieved 28/11/17 from <https://www.svd.se/livsmedelsverket-gift-i-dricksvattet-troligt-i-fler-kommuner> Published in Swedish
12. Taylor, A., A. Glimskär, M. Viketoft, H. Friberg, B. Andersson, M. Jonsson, R. Bommarco, L. Andersson and A. Hedström Ringvall (2015). "*Utformning av miljöövervakningsprogram för biologisk mångfald och skadegörare i och vid åkermark.*" Design of environmental monitoring programs for biodiversity and harmful organisms in and around arable - SLU Sveriges lantbruksuniversitet (Swedish University of Agricultural Sciences). Retrieved 28/11/17 from <http://www.jordbruksverket.se/download/18.59ca4e98158b374159910042/1480491627216/Rapport_%C3%A5kermarks%C3%B6vervakning.pdf>) Published in English (site also in Swedish).

## Natura 2000

1. Lund University (no date). Svensk Fågeltaxering (Swedish Birdwatching). In Monitor the common birds of Sweden. The project is carried out at *the Department of Biology, Lund University, as a part of a national environmental monitoring project run by The Swedish Environmental Protection Agency and supported by all the 21 County Administrative Boards of Sweden.*, English and Swedish. Retrieved 28/11/17 from <http://www.fageltaxering.lu.se/english> . Published in English.
2. SLU Sveriges lantbruksuniversitet (Swedish University of Agricultural Sciences) (no date). Miljödata MVM. In *Environmental Data: Here you can search for soil, water and environmental data that exist within two of SLU's data capabilities, Lakes and Waters, as well as Farmland.* Retrieved 28/11/17 from <http://miljodata.slu.se/mvm/>. Published in Swedish.
3. SLU Sveriges lantbruksuniversitet (Swedish University of Agricultural Sciences) (no date). The Swedish Species Information Centre. In *Accumulate, analyse and disseminate information concerning the species and habitats occurring in Sweden*. Retrieved 28/11/17 from <https://www.artdatabanken.se/en/>. Published in English and Swedish.
4. SLU Sveriges lantbruksuniversitet (Swedish University of Agricultural Sciences) (2017). Environmental Monitoring at SRH (Department of Forest Resource Management). Retrieved 28/11/17 from <https://www.slu.se/en/departments/forest-resource-management/environment/?si=FF6839F203CE3F5CB87E2B05387BD3B9&rid=1530929115&sn=sluEPi6-prodSearchIndex>. Published in English and Swedish.
5. SLU Sveriges lantbruksuniversitet (Swedish University of Agricultural Sciences) (2017). Nationell inventering av landskapet i Sverige, NILS. In *National inventory of the landscape in Sweden, NILS*, English (site also in Swedish), <https://www.slu.se/centrumbildningar-och-projekt/nils/> .
6. Swedish Environmental Protection Agency (no date). The Environmental Objectives Portal. In *The Environmental Objectives Portal is a gateway to information about Sweden's environmental objectives and progress towards achieving them.* Retrieved 28/11/17 from <http://www.miljomal.se/Environmental-Objectives-Portal/>. Published in English and Swedish.
7. Swedish Environmental Protection Agency (no date). Environmental Objectives: Who does what? Retrieved 28/11/17 from <http://www.miljomal.se/Environmental-Objectives-Portal/Undre-meny/Who-does-what/>. Published in English and Swedish.

## Water Framework Directive

1. Andersen, J. H., J. Aroviita, J. Carstensen, N. Friberg, R. K. Johnson, P. Kauppila, M. Lindegarth, C. Murray and K. Norling (2016). "Approaches for integrated assessment of ecological and eutrophication status of surface waters in Nordic Countries." *Ambio* 45(6): 681-691.
2. Artportalen (no date). "*Rapportsystem för växter, djur och svampar.*" Reporting system for plants, plants and fungi. Retrieved 23/11/17 from <https://artportalen.se/> Published in English and Swedish.
3. Fölster, J., R. K. Johnson, M. N. Futter and A. Wilander (2014). "The Swedish monitoring of surface waters: 50 years of adaptive monitoring." *Ambio* 43(1): 3-18.
4. Länsstyrelsen Dalarnas län (no date). "*DPSIR-modellen.*" County Administrative Board of Dalarna County - DPSIR models - referring to inclusion of social aspects of “context” of the socio-ecosystem in monitoring for the Swedish WFD: Since DPSIR conceptual model is built into WFD a range of aspects of context are monitored. Retrieved 24/11/17 from <http://www.lansstyrelsen.se/Dalarna/Sv/miljo-och-klimat/miljomal/Pages/dpsir.aspx> Published in Swedish.
5. Lund University (no date). "*Svensk Fågeltaxering (Swedish Birdwatching).*" Monitor the common birds of Sweden. The project is carried out at the Department of Biology, Lund University, as a part of a national environmental monitoring project run by The Swedish Environmental Protection Agency and supported by all the 21 County Administrative Boards of Sweden. Retrieved 24/11/17 from <http://www.fageltaxering.lu.se/english> Published in English and Swedish.
6. SLU Sveriges lantbruksuniversitet (Swedish University of Agricultural Sciences) (2016). "*Data host for lakes and watercourses.*" SLU are national data hosts for data collected from national and regional fresh water monitoring, as well as from recipient monitoring. Retrieved 24/11/17 from <https://www.slu.se/en/departments/aquatic-sciences-assessment/data-host/> Published in Swedish and English.
7. SLU Sveriges lantbruksuniversitet (Swedish University of Agricultural Sciences) (2016). "*Miljödata MVM.*" Environmental Data: Here you can search for soil, water and environmental data that exist within two of SLU's data capabilities, Lakes and Waters, as well as Farmland. Retrieved 24/11/17 from <http://miljodata.slu.se/mvm/> and <https://www.slu.se/miljoanalys/statistik-och-miljodata/miljodata/webbtjanster-miljoanalys/miljodata-mvm/introduktion/> Published in Swedish and English.
8. SLU Sveriges lantbruksuniversitet (Swedish University of Agricultural Sciences) (2017). "*Agricultural monitoring catchments.*" Agricultural impact on water quality in stream water and groundwater as well as load of nitrogen, phosphorus and pesticides is monitored in a long-time perspective in small catchments dominated by arable land. Retrieved 24/11/17 from <https://www.slu.se/en/research/research-infrastructure/anlaggningar/agricultural-monitoring-catchments/> Published in English and Swedish.
9. SLU Sveriges lantbruksuniversitet (Swedish University of Agricultural Sciences) (2017). "*Environmental Monitoring at SRH.*" Retrieved 24/11/17 from <https://www.slu.se/en/departments/forest-resource-management/environment/?si=FF6839F203CE3F5CB87E2B05387BD3B9&rid=1530929115&sn=sluEPi6-prodSearchIndex> Published in English and Swedish.
10. SLU Sveriges lantbruksuniversitet (Swedish University of Agricultural Sciences) (no date). "*The Swedish Species Information Centre.*" Accumulate, analyse and disseminate information concerning the species and habitats occurring in Sweden. Retrieved 24/11/17 from <https://www.artdatabanken.se/en/> Published in English and Swedish.
11. Swedish Environmental Protection Agency (no date). "*The Environmental Objectives Portal.*" The Environmental Objectives Portal is a gateway to information about Sweden's environmental objectives and progress towards achieving them. Retrieved 23/11/17 from <http://www.miljomal.se/Environmental-Objectives-Portal/> Published in English and Swedish.
12. Swedish Environmental Protection Agency (no date). "*Environmental Objectives: Who does what?*". Retrieved 23/11/17 from <http://www.miljomal.se/Environmental-Objectives-Portal/Undre-meny/Who-does-what/> Published in English and Swedish.
13. Vattenmyndigheterna (no date). "*Vattenmyndigheterna.*" Responsibility for implementation of the water regulation has been delegated to five water authorities. This is the site for the Water Authorities. . Retrieved 23/11/17 from <http://www.vattenmyndigheterna.se/Sv/Pages/default.aspx> Published in.
14. VISS (no date). "*Vatteninformationssystem Sverige.*" WISS (Water Information System Sweden) is a database that has been developed by the Competent Authorities of the Swedish Water Districts, the County Administrative Boards and the Swedish Agency for Marine and Water Management. In WISS are there classifications and maps of all Swedish major lakes, rivers, groundwater and coastal waters. . Retrieved 23/11/17 from <http://viss.lansstyrelsen.se/About.aspx> Published in English and Swedish.
