# Supplementary material for: Data summarizing monitoring and evaluation for three European environmental policies in 9 cases across Europe
Source: Data Brief. 2019 Feb 28;23:103785. doi: 10.1016/j.dib.2019.103785 (PMC6660552; doi:10.1016/j.dib.2019.103785)
Supplement: Multimedia component 3 [file mmc3.docx]

**Appendix B: Summary of policy-driven Monitoring and Evaluation (M&E) practices in each case**

This document summarizes different aspects of M&E practices carried out across 9 geographical cases. Under each heading, a separate table is provided for each of 3 policy areas considered: the WFD (Water Framework Directive); N2K (Natura 2000 network of protected areas); AES (Agri-Environment Schemes under the Common Agricultural Policy). Within each table the practices within each case is summarised in a separate row. The information from which these summaries are derived is provided in appendix A.

Contents

[1. What is monitored? 2](#_Toc536691219)

[1.1. Are monitoring systems able to understand ecosystem processes? 2](#_Toc536691220)

[1.2. Are social and economic aspects of the socio-ecological system monitored? 7](#_Toc536691221)

[1.3. Does monitoring permit understanding of context? 12](#_Toc536691222)

[2. How is monitoring carried out? 18](#_Toc536691223)

[2.1. Are secondary data being used? 18](#_Toc536691224)

[2.2. Are state and non-state actors involved? 19](#_Toc536691225)

[2.3. Are the data publicly available and accessible? 20](#_Toc536691226)

[3. How is monitoring used in decision-making? 22](#_Toc536691227)

[3.1. Are there any publicly documented uses of monitoring data to revise management? 22](#_Toc536691228)

[3.2. Is there public availability of evaluation processes, and any other uses of monitoring information (including in policy)? 27](#_Toc536691229)

Acronyms commonly used within the tables:

| AES | Agri-Environment Schemes – part of CAP |
| --- | --- |
| CAP | European Common Agricultural Policy |
| DPSIR | Driver-Pressure-State-Indicator-Response framework |
| N2K | Natura 2000 (European network of nature protection areas) |
| NGO | Non-Governmental Organisation |
| PoM | Programmes of Measures |
| RBMP | River Basin Management Plans – plans made under WFD, contain PoMs |
| RDP | Rural Development Programme – contain AES, part of CAP |
| WFD | European Water Framework Directive |

# What is monitored?

## Are monitoring systems able to understand ecosystem processes?

Table 1‑1 WFD: Are monitoring systems able to understand ecosystem processes?

|  | **What attributes of ecosystems are monitored (abiotic and biotic, and interactions) and does this allow understanding of ecosystems?** | **Are any examples of redundant info collected but not used/useable?** | **Are there any examples where info relevant to ecosystems is available from other sources, but is not used as secondary data?** |
| --- | --- | --- | --- |
| Catalonia | Range of abiotic (physical-chemical, hydro-morphological) and abiotic (relevant biodiversity components) variables are used to assess status. | Potentially for biological quality, this is evaluated using several indicators. Although there is spatial and temporal variation. | No response. |
| Estonia | Range of abiotic (physical-chemical, hydro-morphological) and abiotic (relevant biodiversity components) variables are used to assess status. The WFD supports an approach where the management of water ecosystems is considered holistically, i.e. at catchment level. To what extent this has been realised in practice is another question. | Not possible to assess based on document analysis. | Not possible to provide examples based on document analysis. |
| Finland | Range of abiotic (physical-chemical, hydro-morphological) and abiotic (relevant biodiversity components) variables are used to assess status as required by WFD. Though this integrates interaction between effects, there is no specific interaction effects assessment. | Yes, as many taxonomic groups respond similarly to common pressures, such as elevated nutrients. Hence, monitoring all taxonomic groups as stipulated by the WFD is probably not justifiable in many situations i.e. in areas with known pressures. | Satellite data is not directly used, or used as secondary data in status assessment. |
| Flanders | The VMM (Flemish Environment Agency) monitoring strategy includes both abiotic and biotic indicators (± 100 different attributes). Interactions are not explicitly considered in the monitoring schemes, but interpretations are made in reports and feed site specific PoMs. | Not applicable. | Probably not. |
| Hungary | Important aspects of water abiotic features are monitored along with several biotic indicators. The system and the spatial resolution allows an understanding of the interactions. | Not answerable, as not enough familiarity with the policy area. | Habitat mapping (N2K) could be high relevance but is not currently used. |
| Romania | Both biotic and abiotic indicators are monitored according to the requirements of WFD, and they could allow for identifying at least some interactions. Compared to Northern and Western Europe, biological indicators for Eastern European water systems are less developed. | Not aware of any examples. | Information collected for air and soil quality as well as those provided under N2k are relevant for understanding water ecosystem state. No awareness of how used to redesign PoMs. |
| Scotland | SEPA (Scottish Environment Protection Agency) monitoring strategy includes both abiotic and biotic indicators. Interactions are not considered in the monitoring, or very limited. Indicators (UK) are aligned by scientific disciplines rather than by systems. | No examples, but as in other countries the wide range of monitoring data is not always directly used. | No evidence available. |
| Slovakia | Important aspects of water abiotic features are monitored along with several biotic indicators of ecological status and potential. Interconnections of monitoring results and relationship to policies are assessed. | Not aware of any examples. | No evidence available. |
| Sweden | A comprehensive set of abiotic and biotic variables are monitored in representative water bodies. Within water bodies it provides understanding of ecosystems, expert view is that flow of material and energy between water bodies not included. | Yes, as many taxonomic groups respond similarly to common pressures, such as elevated nutrients. Hence, monitoring all taxonomic groups as stipulated by the WFD is probably not justifiable in many situations i.e. in areas with known pressures. | No evidence available. |

Table 1‑2 N2K: Are monitoring systems able to understand ecosystem processes?

|  | **What attributes of ecosystems are monitored (abiotic and biotic, and interactions) and does this allow understanding of ecosystems?** | **Are there any examples of redundant info collected but not used/useable?** | **Are there any examples where info relevant to ecosystems is available from other sources, but is not used as secondary data?** |
| --- | --- | --- | --- |
| Catalonia | None, the focus of monitoring is on species and habitats. Monitoring habitats could be seen as reflecting ecosystem function, to a limited degree. | No examples. | Forest biophysical variables maps, and biodiversity data from bird and butterfly databases are available but not used. |
| Estonia | Biotic and abiotic factors are monitored, but different elements are covered in sub-programmes. With the exception of some monitoring programmes (forest/lake ecosystems) understanding of ecosystems is limited by a lack of integration. | Not known. | Not known. |
| Finland | Several biotic and abiotic factors are monitored; however, this does not necessarily generate understanding of ecosystems. | No examples. | No examples, most primary and secondary data seems to be used. |
| Flanders | Only biotic data is collected. To assess condition of habitats, key species and indicator species are monitored as proxy for abiotic measures. | No examples. | 1) A conservation NGO's species observation website (*Natuurpunt*). 2) a network of piezometers in nature reserves (measuring groundwater levels) |
| Hungary | No abiotic indicators are monitored. | No examples. | Examples are: Monitoring programme of the Hungarian Ornithological Society ‘MME’ (collecting land use + habitat types of sampling sites), CLC land cover (changes) data. |
| Romania | Both biotic and abiotic. Under the 'threats and pressures' heading some interactions are assessed, but very limited. | Unknown. | Monitoring data on water and air quality, meteorological and climatic data. |
| Scotland | Site Condition Monitoring includes both biotic and abiotic features (like earth science & geomorphological features). Webpages suggest focus on individual features, unclear to what extent interactions are analysed. | No examples. | More landscape data could be used to achieve better integration of indicators (landscape monitoring programme). |
| Slovakia | Both biotic and abiotic. Abiotic ecological factors essential for species included. Biotic include species composition in forests. Ecological-functional zones (EFZ) are the tool for spatially assessing species-habitats interactions. | No examples. | Other relevant data is not considered as secondary data. |
| Sweden | Yes, interactions between habitats and species allow understanding of ecosystems and their change over time. Various monitoring programmes contribute (see template for details of N2K monitoring in Sweden and DPSIR). | No examples. | No examples, wide range of primary and secondary data is used. |

Table 1‑3 AES: Are monitoring systems able to understand ecosystem processes?

|  | **What attributes of ecosystems are monitored (abiotic and biotic, and interactions) and does this allow understanding of ecosystems?** | **Are any examples of redundant info collected but not used/useable?** | **Are there any examples where info relevant to ecosystems is available from other sources, but is not used as secondary data?** |
| --- | --- | --- | --- |
| Catalonia | Limited in to habitat types included in the ‘SIGPAC’ (*Sistema de Información Geográfica de Parcelas Agrícolas).* | No redundant information is collected. | Some secondary data of N2k case (e.g. on land cover maps, and bird or butterfly abundance models) could be used in AES. |
| Estonia | A range of abiotic and biotic indicators are collected that cover a range of domains e.g. soil and water. Assessment of interactions is not a focus, though providing some insight into the understanding of ecosystems status and its causal linkage to specific AES. | No example found | None to our knowledge (difficult to assess based on document analysis). |
| Finland | A wide range of biotic attributes related to fungi, plant, insect and animal species is collected under MaaMET monitoring. | No clear cut evidence of redundant information, a possible example related to nitrogen monitoring was provided. | An example of data from third parties is Birdlife Finland^[[1]](#footnote-1)^, this could be used more efficiently as secondary data. |
| Flanders | Monitoring AES is about biotic data e.g. species rich grassland indicator species, with no abiotic indicators measured. | Not applicable. | Yes, two examples are: The biggest nature conservation NGO in Flanders (Natuurpunt), having thousands of records of all kinds of organisms; and a network of piezometers throughout Flemish nature reserves to measure groundwater levels year-round. |
| Hungary | Abiotic information on nutrient (N and P) balances and load to groundwater. This provides some level of ecosystem understanding. | No example found. | No, the most relevant secondary data are used. |
| Romania | A wide range of abiotic e.g. fertiliser inputs and energy use, as well as biotic information e.g. grassland community structure and farmland bird surveys. No evidence of an assessment of their interaction. | No example found. | A range of relevant information on climate variability, air quality data as well as scientific studies are available, but are not used as secondary data. |
| Scotland | The main focus is on biotic attributes e.g. invertebrate, plant and bird species, with limited abiotic information on landscape features are collected. An example of evidence that interactions are monitored are beetle counts- an important food source for farmland birds. | No example found. | Potentially relevant (secondary) data for designing schemes for farm ecosystems would be the nutrient levels in soil and water bodies, and more interestingly the potential impact of AES on these. |
| Slovakia | Reference was made to the collection of abiotic indicators e.g. to delineate LFAs based on soil quality, but unsure whether actually applied. An example of supporting HNV (High Nature Value farming) leading to less land abandonment was provided. | No example found. | Yes, climate data are available but are not used for monitoring or evaluation of measures regarding to measures related to "contribution to combating climate changes". |
| Sweden | A range of biotic and abiotic attributes are collected using a randomised sampling design (ca.500 sites/six year interval). However, given the heterogeneous nature of agriculture landscapes, this programme does not allow systematic understanding of ecosystems. A number of spatially restricted research projects provide a better understanding of pertinent ecosystem processes. | Not aware of any redundant information being collected. | No example found. |

## Are social and economic aspects of the socio-ecological system monitored?

Table 1‑4 WFD: Are social and economic aspects of the socio-ecological system monitored?

|  | **What social or economic information is collected under formal monitoring programmes?** | **Does any of this information allow understanding of interactions between humans and their natural environment?** | **Describe examples of potentially-relevant social or economic info collected for related national policies that is not currently used as 2ery data for M&E of above?** |
| --- | --- | --- | --- |
| Catalonia | Very scarce, focus is on status of water bodies including priority substances. | Some references e.g. alternation of water regimes, and water provision for the citizenship. | National and regional statistic services (e.g. ‘IDESCA’T) can provide relevant social and economic info at county/ municipality level (e.g. population, unemployment, GDP, etc.). |
| Estonia | Probably none directly. | No, since none collected. | No evidence available. |
| Finland | The only economic information collected is household water consumption, no social info is collected. | No, since limited economic information is collected. | House hold water consumption is not used as secondary data for monitoring and evaluation |
| Flanders | No primary data collection dealing with social and economic items is used to implement the WFD, but secondary data regarding infrastructure construction are sometimes consulted. | No, since no formal monitoring of social or economic factors. | No evidence available. |
| Hungary | There is no monitoring of social or economic issues. | None. | AES CAP areas with low input nutrients. |
| Romania | Though the National Water Management Plan (NWMP) refers to social and economic aspects, no clear example of formal monitoring was found. | Yes, information on anthropogenic pressures should be collected. | National Institute of Statistics collect and make synthesis about different indicators e.g. land use, productivity in different economic sectors, population info.  Unclear whether this information is used in water management e.g. economic analysis of water use. |
| Scotland | No clear example found of monitoring of social issues. Though actual monitoring may differ to what written down in monitoring strategies. | No, as there is nothing collected. | No clear examples found. |
| Slovakia | No social or economic data are collected under formal monitoring program, but the Water monitoring data are sometimes included to evaluate social impact of flooding, and economic analysis of water use. | No, as there is nothing collected. | Socio-economic indicators including GDP could be used to assess water utilization. |
| Sweden | Seldom used in WFD with the exception of heavily modified water bodies. | Not really, as only used with heavily modified water bodies. | No evidence available. |

Table 1‑5 N2K: Are social and economic aspects of the socio-ecological system monitored?

|  | **What social or economic information is collected under formal monitoring programmes?** | **Does any of this information allow understanding of interactions between humans and their natural environment?** | **Describe examples of potentially-relevant social or economic info collected for related national policies that is not currently used as 2ery data for M&E of above?** |
| --- | --- | --- | --- |
| Catalonia | Not much. The ministry does mention something on aligning habitat management to community interests, but unclear whether really put to practice. | No, although some academic research on provision and demand of ecosystem services is done. | Cartography of ecosystem services is available, but not being used. |
| Estonia | None under formal policy, although some socio/economic factors are considered in management plans. | Probably not, but difficult to tell. | Some state agencies arrange monitoring which could be interpreted as considering socio-economic aspects, e.g. the State Forest Management Centre (responsibilities include recreation management on protected areas) conducts visitor monitoring on Protected Areas. These data are used for compiling management plans. |
| Finland | Some socio/economic information from pressures and trends (e.g. forestry, agriculture, urbanisation). Stakeholders are heard in targeted evaluations (see template). Employment and income impacts are evaluated. | Yes, but ambiguous. | No example available. |
| Flanders | Not much. Since 2016, impact of N-emissions by farms near N2K sites is being assessed | Yes, but ambiguous. | No example. |
| Hungary | No socio/economic information is collected directly, but 'threatening factors' and land use change are considered in the Landscape Monitoring of the National Biodiversity Monitoring System (NBmR) – III Project | Evaluation of habitats and land-use change allows insight in socio/economic interactions with the system, e.g. intensification of agriculture; land/grassland abandonment; urbanization; higher habitat pressure by tourism; expanding biotic invasion; restoration efforts; land drying effect etc. | Central GIS database on AES under CAP, National Forest Management Database, and the National Game Management Database could be used, but are usually not used. A newly started project aims at using this data as secondary data. |
| Romania | Socio-economic context is part of the management plans, as are threats and pressures - including anthropogenic and natural ones. Still in early stages, first management plans are currently in process of approval. | Yes, that would allow for some understanding of human influences and interactions on a site. | Monitoring info collected for RDP (AES) related to forestry, water management in agriculture |
| Scotland | Pressures, including social ones, that might change the status are also evaluated, like over-grazing, recreation, agricultural operations, development, or trampling. | Yes, human activity that is likely to affect the site adversely is considered. | No example. |
| Slovakia | Historic area use, socio/economic importance of area. 11 sub-chapters include nature protection, forestry, agriculture, transport, tourism, hunting, mining, utilization of water, education, research, other utilization. Need to link socio-economic with habitat/species data. | For particular N2K areas – creation of the model of optimal care (maintenance) for habitats or habitats for species with same parameters of utilization, which will reflect value of favourable status – the sustaining model , the regeneration model. Creation of ecologic-functional areas and zones. | Environmental Impact Assessments |
| Sweden | Social/economic aspects not considered by national or regional authorities, but some by research (see template for papers). | Interactions focus on human-carnivores and conflicts between habitats and project development (e.g. railway). | No example. |

Table 1‑6 AES: Are social and economic aspects of the socio-ecological system monitored?

|  | **What social or economic information is collected under formal monitoring programmes?** | **Does any of this information allow understanding of interactions between humans and their natural environment?** | **Describe examples of potentially-relevant social or economic info collected for related national policies that is not currently used as 2ery data for M&E of above?** |
| --- | --- | --- | --- |
| Catalonia | A limited amount of information in the Unique Agrarian Declaration (DUN) on economic and social aspects related to land owners is provided along with technical information on land use practices. | Yes, land use is how land managers interact with their land. | Other payment schemes (e.g. Basic payment and Payment to young farmers) collect some more info on the socio-economic status of farmers, which could be used for AES. |
| Estonia | A range of social and economic information is collected as part of the Estonian RDP under the themes of income for rural enterprise, share of organic products e.g. amount of products produced, and env awareness. | Probably not, although some info on socio-economic as well as environmental aspects are collected, interactions seem not to be the focus here (difficult to answer based on document analysis). | The social and economic information collected under the Estonian RDP. |
| Finland | Information about how agri-environmental support has affected the potential for farming. | Yes, understanding of agri-environmental support impact on farming helps to understand how agri-environmental support should be developed to increase its impact. | No example of unused relevant data. |
| Flanders | Social and economic information is monitored (though not formally through the AES) and used in annual Rural Development report to the European Commission. | Indirect information is collected on the nature of farm businesses and farmer social circumstances e.g. successor or not. | No example of unused relevant data. |
| Hungary | Though no direct monitoring, there is impact monitoring to reveal the influence of management treatments by specific AES aim. | No redundant information is collected. | A range of databases e.g. CORINE Land Cover are used for evaluation. |
| Romania | A range of social e.g. training level of farmer and their age, and economic information e.g. about the farm and its operation are collected. | Land management applied at farm level is an example of this information. | An example of environmental policy on hazardous substances e.g. the import and use of these is collected by National Agency for Environmental Protection and (ii) used as secondary information. |
| Scotland | Under the Scottish AES limited social information was collected for evaluation of previous schemes, this included one-off survey of farmers perceptions of the AES. | The one-off survey would to a limited extent. | A range of potentially-relevant social e.g. farmers views on biodiversity or economic information e.g. farm income and size is collected. It is not clear if this is used for monitoring and evaluation. |
| Slovakia | The annual implementation reports draw on an Operational Database Of Agricultural Paying Agency that contains information on the nature of the farm business and its operations. | Land use related information e.g. managed areas in LFAs were provided as examples. | It was suggested that the annual reports and evaluation reports could be used to redesign the AES measures to stop land abandonment in LFAs. |
| Sweden | Apart from standard EU reporting requirements e.g. on farm business and their operation there is little formal monitoring. A range of research projects have been carried out. | In line with other countries, land use information provided some understanding. | Periodic synthesis activities have assessed the impact of CAP on the environment. Unclear what social or economic information was collected for this purpose. |

## Does monitoring permit understanding of context?

Table 1‑7 WFD: Does monitoring permit understanding of context?

|  | **What aspects of context are monitored (social, technical, env, economic, policy)?** | **Are there any examples of redundant info collected but not used/useable?** | **Are there any examples where info relevant to ecosystems is available from other sources, but is not used as secondary data?** |
| --- | --- | --- | --- |
| Catalonia | Limited information on environmental and socio-economic (e.g. water provision per capita) is provided at the water body level and summarised at catchment and hydrological district scales. | No redundant info is collected. | National and regional statistic services (range of social and economic info at catchment and district levels) from county/ municipality databases. |
| Estonia | The official regular monitoring programme probably does not focus on any contextual aspects directly, but some related aspects are considered when compiling the water management plans, e.g. by outlining the pressures and impacts as stated in the Water Act. | No example found. | None to our knowledge (difficult to assess based on document analysis). |
| Finland | Environmental, technical, and economic aspects are monitored whereas no social or policy aspects are monitored. | Policy integration across different ministries led revised monitoring in 2006, were non WFD essential monitoring was stopped. | A wide range of other monitoring programs, (e.g. listed in N2K), but these do not contribute to RBMP. |
| Flanders | Some social and environmental aspects may be monitored. | Not applicable. | Yes, the website of the biggest nature conservation NGO in Flanders (Natuurpunt), containing thousands of records of all kinds of organisms, including water-related plants, insects, birds. |
| Hungary | Monitoring is not really focussing on contextual aspects. | No redundant info is collected. | Yes, Results of N2K landscape level habitat mapping: Spatial and quality/naturalness features and water relations of these habitats and degrading, threatening factors. |
| Romania | A range of social e.g. population, technical e.g. Quality of Surface Waters Required for Protection and Improvement in Support of Fish Life (from 28.02.2002), environmental e.g. variables related to climate change, soils and N2K monitoring, economic e.g. GDP are monitored. No example of policy context was found. | No example found. | No example found. |
| Scotland | Some monitoring of environmental context e.g. proximity of farmland. | No example found. | SEPA (Scottish Environment Protection Agency) does have Supporting Guidance (WAT-SG-67) for Assessing the Significance of Impacts - Social, Economic, Environmental. This suggests that such data is available, but it is unknown if this is actually collected, by whom, or if this is used as secondary data in monitoring. |
| Slovakia | A wide range of aspects of context are monitored that include environmental e.g. pollution, and technical e.g. how samples and data are treated. | No example found. | No example found. |
| Sweden | Since DPSIR conceptual model is built into WFD, a range of aspects of context are monitored.  However, WFD monitoring is solely focused on assessing the physiochemical and biological quality of the water body and, to date, this is being done by county administrative boards, with little interaction with stakeholders. | No example found. | No example found. |

Table 1‑8 N2K: Does monitoring permit understanding of context?

|  | **What aspects of context are monitored (social, technical, env, economic, policy)?** | **Are there any examples of redundant info collected but not used/useable?** | **Are there any examples where info relevant to ecosystems is available from other sources, but is not used as secondary data?** |
| --- | --- | --- | --- |
| Catalonia | Some context monitoring is mandatory (e.g. human activities around sites, see template), but implementation is questionable. | No examples. | The Barcelona Province Council provides some context information. |
| Estonia | Context not systematically monitored. Context is considered in management plans. Main objective of official policy is environmental. | No examples found. | No examples found. |
| Finland | Context not systematically monitored. Context is considered in management plans. | No examples. | Finland has a huge reservoir of monitoring data that are drawn upon in evaluations, but not systematically. |
| Flanders | None, drivers/context are neglected, a singular focus on biotic data. | No examples. | A conservation NGO's species observation website (natuurpunt). |
| Hungary | Factors threatening habitats are recorded, i.e. mining, pollution, various management options, and biological invasions, interactions, environmental and natural disasters. | No examples. | No examples. |
| Romania | Very limited, and only in management planning rather than formal monitoring. Contextual data does not appear in reports. | No examples found | No examples found |
| Scotland | Pressures are identified, including contextual indicators like invasive species, water management, extraction (quarrying), infrastructure, agriculture, pollution etc. N2K monitoring data itself is also used as secondary data for wider policy objectives (see template for examples). | No examples | No examples |
| Slovakia | All aspects of context considered, see template for examples. | No examples. | There is relevant information that is currently not used as secondary data, such as the CBD (Convention on Biological Diversity) indicators and surrogates. |
| Sweden | Using the DPSIR framework, for NK2 sites it is stressed that S (status) and I (impacts) are the main focus, while P (pressures) is used when selecting sites and when evaluating status and trends. | No examples found. | No examples found. |

Table 1‑9 AES: Does monitoring permit understanding of context?

|  | **What aspects of context are monitored (social, technical, env, economic, policy)?** | **Are there any examples of redundant info collected but not used/useable?** | **Are there any examples where info relevant to ecosystems is available from other sources, but is not used as secondary data?** |
| --- | --- | --- | --- |
| Catalonia | Socio-Economic (owners), environmental (habitat types) and technical (cropping systems) aspects of context are monitored. | No redundant info is collected. | No example found. |
| Estonia | A wide range of landscape e.g. landscape elements and social and economic aspects e.g. income for rural enterprises. | No example found. | No example found. |
| Finland | A range of studies into social, technical, env, economic, policy contexts have been carried out, it is not clear if these are monitored as part of the formal monitoring policy. | Potentially monitoring related to nitrogen maybe redundant, but this is not 100% clear. | Yes, secondary data from third parties such as the Birdlife Finland <https://www.birdlife.fi/in-english/> or companies delivering abiotic data to national registers, which could be used more efficiently. |
| Flanders | Information on social, economic and environmental context is available, though the focus of AES monitoring is biotic information. | Not applicable. | Yes, there are multiple examples e.g. social and economic information on farms and farmers is available and could be used more to understand potential impacts on the system. |
| Hungary | A range of environmental context aspects e.g. nutrient load to surface waters are monitored in relation to AES measures. A wider set of social and economic information is available for analysis. | No redundant info is collected. | No example found. |
| Romania | A wide range of social e.g. education, technical e.g. land drainage, environmental e.g. conservation status of areas, economic e.g. young farmers business plans supported context information is monitored. | No example found. | Not known. |
| Scotland | There is evidence of technical e.g. targeted support for slurry stores, and environmental context e.g. use of remote sensing to assess land use change. | No example found. | Potential other sources available, like climate data. |
| Slovakia | Socio-Economic (public expenditure, type and size of farm holders), environmental (habitat types) and technical (cropping systems, management practices) aspects of context are monitored. | No example found. | Climate data are available but are not used for monitoring or evaluation of measures related to combating climate change. |
| Sweden | No clear evidence of context being monitored. | No example found. | No example found. |

# How is monitoring carried out?

## Are secondary data being used?

Table 2‑1 WFD: Are secondary data being used?

|  | **Are secondary data being used?** |
| --- | --- |
| Catalonia | Mainly primary, secondary data only marginally used in monitoring, e.g. biodiversity data. |
| Estonia | Mainly primary, some secondary e.g. statistical body (see original template). |
| Finland | No. |
| Flanders | Mainly primary data, but also secondary data like soil erosion mapping and 'biological evaluation' maps. |
| Hungary | No. |
| Romania | This is not clear from publicly available material. |
| Scotland | Mainly primary, some secondary data, e.g. citizen science (anglers monitoring biological quality/ obstacles to fish migration etc.). |
| Slovakia | Mainly primary, some secondary includes protected areas bordering with water bodies. |
| Sweden | Majority is primary. Secondary data includes e.g. land use, airborne pollutants, and invasive species. |

Table 2‑2 N2K: Are secondary data being used?

|  | **Are secondary data being used?** |
| --- | --- |
| Catalonia | Yes, N2K monitoring uses mainly secondary data, from private associations although funded by state. E.g. habitat cartography, bird status etc. |
| Estonia | Not much secondary data, although some from national environmental monitoring programme. |
| Finland | Mainly primary, but also secondary. |
| Flanders | Mainly primary data, but also habitat mapping. |
| Hungary | No secondary data. |
| Romania | No secondary data. |
| Scotland | Yes, some secondary data e.g Seabird 2000 and Wetland Bird Survey (WeBS). |
| Slovakia | Primary only. |
| Sweden | Both primary and secondary. Example of secondary data: land use data, climate data etc. to estimate threats on habitat/species. |

Table 2‑3 AES: Are secondary data being used?

|  | **Are secondary data being used?** |
| --- | --- |
| Catalonia | Currently no secondary data is used, although it would be useful. |
| Estonia | Mainly primary, although some secondary, e.g. national statistics. |
| Finland | Both primary and secondary data are used. |
| Flanders | Only primary data. |
| Hungary | Both. Secondary data includes forest management-, game management-, and land cover databases. |
| Romania | Both. Secondary data includes satellite images for compliance checks, and modelling, soils quality. |
| Scotland | Mainly primary data is used from non-state evaluation teams, it is unclear whether secondary data is used. |
| Slovakia | Both. |
| Sweden | Both. |

## Are state and non-state actors involved?

Table 2‑4 WFD: Are state and non-state actors involved?

|  | **Are both state + non-state agencies providing monitoring data?** | **Is citizen science being used for monitoring?** |
| --- | --- | --- |
| Catalonia | State-led, parts are outsourced. | Marginally, some species (mainly birds and butterflies). |
| Estonia | State-led, parts are outsourced. | No. |
| Finland | State-led, parts are outsourced. | No. |
| Flanders | State agency. | No. |
| Hungary | Only state agency: general directorate of water management. | No. |
| Romania | Only state agency. | No. |
| Scotland | Only state agency. | Citizen science developed for other purposes is used as secondary data for WFD monitoring (but constitutes very small part of monitoring programme). |
| Slovakia | State-led monitoring, mainly state institutions. | No. |
| Sweden | Both state and non-state agencies. | Much of the information stored by the Swedish Species Information Centre is provided by citizen science. |

Table 2‑5 N2K: Are state and non-state actors involved?

|  | **Are both state + non-state agencies providing monitoring data?** | **Is citizen science being used for monitoring?** |
| --- | --- | --- |
| Catalonia | Both. | Yes, in data collection, mainly birds but also butterflies (some initiatives on plants, still local). |
| Estonia | Both. | Yes, not primarily, but supplemented by verified data from volunteers. |
| Finland | Both. | Yes (including volunteers) |
| Flanders | Both. | Volunteers at NGO. |
| Hungary | Both. | Yes, for birds. |
| Romania | Both, through subcontractors. | Yes, both directly (through on-line platforms) and indirectly (as volunteer at NGOs). |
| Scotland | Both, although unclear how. | Yes. |
| Slovakia | Both. | Yes, volunteers and non-experts are involved. |
| Sweden | Both, although for the Habitats Directive it is more using state agency, while for the Birds Directive it is mainly citizen science. | Yes, especially for the Birds Directive. |

Table 2‑6 AES: Are state and non-state actors involved?

|  | **Are both state + non-state agencies providing monitoring data?** | **Is citizen science being used for monitoring?** |
| --- | --- | --- |
| Catalonia | Only state agency. | Yes, but not always considered in directive monitoring as secondary data. |
| Estonia | State and academia. | No. |
| Finland | Both state and non-state. | No. |
| Flanders | Both, including birds surveys by NGOs. | No. |
| Hungary | Both, especially birds by non-state agencies. | Marginally, for bird surveys. |
| Romania | Both state and non-state. | Marginally, for bird surveys. |
| Scotland | Non-state evaluation team- tendered by the government, and academia. | No. |
| Slovakia | Both state and non-state. | Unknown. |
| Sweden | Both, specialist NGOs contribute. | Marginally, for bird surveys, and only as secondary data. |

## Are the data publicly available and accessible?

Table 2‑7 WFD: Are the data available and accessible?

|  | **Is there a “data sharing policy” for monitoring data?** | **Please confirm and describe data availability.** | **Please confirm and describe data accessibility/user-friendliness.** |
| --- | --- | --- | --- |
| Catalonia | Open and transparent. | Good. | Good. |
| Estonia | There is no official data sharing policy regarding monitoring specifically, but data is provided via specific monitoring websites, an information system and regular reports. | Good. | Good. |
| Finland | Very open and transparent. | Excellent, both raw data and classification and analysed data. | Good, user-friendly. |
| Flanders | Data are provided upon request, some of them are available in reports on the Environmental agency’s website (VMM). | Good. | Not always user-friendly: understanding the data requires expertise. |
| Hungary | No (not aware of). | Good, new web interface. | Not good for wider public: only flood risk and inundation risk maps are easily accessible. |
| Romania | By law, data should be provided upon request. | Not good - requires requests to obtain data; data is not easily given. | Not good, some synthesis available, but not much data. |
| Scotland | No (not found). | Good. | Good, with a variety of user-specific visualisations. |
| Slovakia | Yes, mostly statistics online. Field data based on law are available on request. | Reasonable. Most is available, some data only commercial and expensive. | Good, user-friendly interface. |
| Sweden | Very open and transparent. | Excellent: raw data, classification and analysed data. | Excellent (except for 'polluter pays' data). |

Table 2‑8 N2K: Are the data available and accessible?

|  | **Is there a “data sharing policy” for monitoring data?** | **Please confirm and describe data availability.** | **Please confirm and describe data accessibility/user-friendliness.** |
| --- | --- | --- | --- |
| Catalonia | Not really. Data are provided upon request. | Intermediate, good for experts, less for citizens. | Intermediate, not always user-friendly. |
| Estonia | Yes, there are some elements of such policies in the form of departmental prescriptions or/and good practices. | Good, variety of visualisations and level of detail. | Good, variety of visualisations and level of detail. |
| Finland | Very open and transparent. | Good. | Very good, a recently developed service for open access sharing exists <https://www.laji.fi/>. |
| Flanders | Not yet, discussion is ongoing about this. | Good. | Good. |
| Hungary | No. | Not good. There are some databases, but only partial operational. | Mixed. Gov: Not good. Best data is found on EU websites, not national ones. NGO’s: better data presentation. |
| Romania | No. | Not good, not freely available, no raw data. Some synthesis reports are available. | Not good. Best data is found on EU websites, not national ones. |
| Scotland | No, not found. | Good. | Good, variety of visualisations. |
| Slovakia | Yes. All data should be available to the public, except for sensitive data. | Good. Raw data available for experts/evaluators. | Good. |
| Sweden | Yes, all is publicly available, except endangered species. | Good. | Good, although raw data sometimes not designed for public. |

Table 2‑9 AES: Are the data available and accessible?

|  | **Is there a “data sharing policy” for monitoring data?** | **Please confirm and describe data availability.** | **Please confirm and describe data accessibility/user-friendliness.** |
| --- | --- | --- | --- |
| Catalonia | No. By law, data should be provided upon request. | Not good, data sharing limited by owners’ privacy. | No response. |
| Estonia | There is no official data sharing policy regarding monitoring specifically, but data is provided via specific monitoring websites, an information system and regular reports. | Reasonable, both raw and analysed data available. | Reasonable, not always adapted to lay readers. |
| Finland | No. | Moderate, but Improving. Species info is available on one website. | Moderate, mainly aimed at experts, not citizens. |
| Flanders | No. | Not good. No raw data. | Not good, spread out over departments/institutes. |
| Hungary | No. | Bad. Raw data not available for public. | Mixed. Government generally bad. Ornithological society data is accessible. |
| Romania | Yes, there is data sharing in some degree between actors for RDP (through Monitoring Committee)^[[2]](#footnote-2)^ and for AES (through the Environment Working Group)^[[3]](#footnote-3)^. | Not good. Mainly administrative and financial synthesis, no raw data. | Not good, only fact sheets and budget allocations, no raw data. |
| Scotland | No. | Not good. SRDP in general: yearly, but no raw data (mainly financial). RPID inspections: not available. AES: 6-yearly evaluation available, no raw data. | Data accessibility is not good: data is difficult to find. |
| Slovakia | No. | Not good: data is available only to experts, not to the public. | Reasonable, reports and statistics available online. |
| Sweden | Yes, although data are limited. | Reasonable, although spread out over many institutes and not recognizable as CAP monitoring. | Not good, spread out over many institutes and not recognizable as CAP monitoring. |

# How is monitoring used in decision-making?

## Are there any publicly documented uses of monitoring data to revise management?

For WFD, the focus of management is programmes of measures (PoMs) for water bodies; For N2K, the focus of management are protected areas; For AES, the focus of management is the choice and design of Agri-Environment Schemes.

Table 3‑1 WFD: Does monitoring inform decisions to revise management?

|  | **Are the management actions updated or changed?** | **How are monitoring data used to make these changes?** | **Are decisions hindered by lack of data?** |
| --- | --- | --- | --- |
| Catalonia | Yes, the programmes of measures are reviewed and approved every 6 years. | Unclear, although the review of the PoMs can drive changes of the metrics. | No evidence available. |
| Estonia | Yes, the management plans are updated as required by the WFD implementation scheme, over the 6-year period. | This is not so clear, the data may be used, but to what extent and how, is not possible to evaluate based on document analysis. | No evidence available. |
| Finland | Yes, the programmes of measures are reviewed and approved every 6 years. However, there can be long lags between actions taken and responses in the receiving waters. | Progress made is documented in national reports and PoM as well as monitoring is adjusted. | No evidence available. |
| Flanders | Monitoring data is used to revise and adjust RBMPs and new or adjusted measures are reported on the website. | it is not clear how these adjustments relates to monitoring, because there are few explicitly written links between the data (reports) and adjusted or new measures. | No evidence available. |
| Hungary | Yes, the revision of the third RBMP was completed in 2015. | The trends to higher water levels had led to new measures to control flows. | No evidence available. |
| Romania | Romanian National River Basin Management Plan was updated and new measures are being implemented in the period 2016-2021, based on the monitoring of the implementation of the measures in the National Management Plan (NMP) approved in 2011. | Yes, the monitoring data are used to make the changes and this is explicitly mentioned in the law. | Lack of data or limited confidence in the data was mentioned in the first NRBMP, under the “problems and uncertainties” chapter. Lack or insufficient data are also mentioned in the literature (see Risnoveanu et al. 2017). |
| Scotland | Yes, the management plans are updated as required by the WFD implementation scheme, over the 6-year period. | The new Scotland RBMP acknowledges how information from environmental monitoring programmes, helped understand pressures better (but there is no evidence that shows how this helped to select the measures). | No evidence available. |
| Slovakia | The management plans for watersheds were elaborated in 2009, and then updated in 2015. | This is not clear, maybe, but the extent is unknown. | No evidence available. |
| Sweden | Yes. The WFD requires updates (every six years) on RBMPs. County administrative boards need to describe what measures are being taken to achieve good ecological status. | Yes. Data are used to assess impacts and trends, and to design and implement programs of measures. | No evidence. However we lack understanding of how multiple pressures effect aquatic systems; and how management interventions function at larger spatial scales. |

Table 3‑2 N2K: Does monitoring inform decisions to revise management?

|  | **Are the management actions updated or changed?** | **How are monitoring data used to make these changes?** | **Are decisions hindered by lack of data?** |
| --- | --- | --- | --- |
| Catalonia | As there are some Spanish N2K areas without management plans, it is too early to answer this yet. | Not answerable as management and monitoring is only applied to a very little proportion of sites. | Not answerable as management and monitoring is only applied to a very little proportion of sites. |
| Estonia | Yes, the management plans for Natura areas are periodically reviewed and updated (normally every 5 years). | There is no information publicly available on how monitoring data is used for redesigning the management plans. | No evidence available. |
| Finland | Steps to ensure of favourable conservation status is required by law. | The systematic uptake of monitoring results is rare; some examples exist but often with long time lags. | No evidence available. |
| Flanders | As the integrated management plans for N2K-sites are still in a start-up phase, it is too early to answer this yet | There have been several changes to the monitoring systems, combined with new management plans, so it is too early to answer this question. | Not applicable. |
| Hungary | As there are some Hungarian N2K areas without management plans, it is too early to answer this yet. | Whilst the plans should consider monitoring data in theory. | Not applicable. |
| Romania | Steps to ensure favourable conservation status is required by law. | The monitoring data is used to inform future actions. | The lack of data is noted as a problem especially when dealing with occurrence, distribution and reference conditions for characterising habitats and species of community interests, especially when historic data are missing. |
| Scotland | Steps to ensure favourable conservation status is required by law. | Site Condition Monitoring is used to inform discussions and decisions between land managers and Scottish Natural Heritage on remedial actions where necessary. | No explicit mention was found of lack of data or evidence that this is hindering management decisions. |
| Slovakia | Management plans for habitats and species are updated and changed. | The monitoring data are used for this purpose. | The lack of data is a problem. Only few species or protected areas have accepted management plans. |
| Sweden | Management plans are updated but current measures are considered inadequate. | Yes. Monitoring data are used to develop and revise action plans; mostly focused on birds, endangered species and their habitats. | No evidence available. |

Table 3‑3 AES: Does monitoring inform decisions to revise management?

|  | **Are the management actions updated or changed?** | **How are monitoring data used to make these changes?** | **Are decisions hindered by lack of data?** |
| --- | --- | --- | --- |
| Catalonia | AES schemes are regularly redesigned (± every 7 year, CAP-period). | Changes usually respond primarily to socioeconomic reasons, and very rarely to conservation issues. | No evidence available. |
| Estonia | The goal of the annual implementation report is to raise the quality of implementation. | Unclear to what extent and how exactly monitoring results affect redesigning the measures | No evidence available. |
| Finland | Yes, there has been increased funding and more focus on biodiversity in the current schemes. | Clearly monitoring results have influenced redesign of AES measures, e.g. the new project called [MYTTEHO](https://www.luke.fi/en/projects/mytteho/) is assessing cost-effectiveness of AES measures. | No evidence available. |
| Flanders | AES schemes are regularly redesigned (± every 7 year, CAP-period). | Monitoring data is used for this partly, but specific research and experience is also used. | No evidence available. |
| Hungary | AES schemes are regularly redesigned (± every 7 year, CAP-period). | No clear evidence for using monitoring data in updating AES. | No evidence available. |
| Romania | AES-CAP schemes are updated / redesigned. | No clear evidence for using monitoring data in updating AES. | The lack of data was not noted at the farm, regional or national level. |
| Scotland | AES-CAP schemes were updated / redesigned, compared with 2007-2013 programme. | Yes, although the data may not be adequate. | Due the aggregated nature of the data, the evaluation team was unable to analyse the management interventions as fully as required. |
| Slovakia | AES-CAP schemes have been updated with some new conditions added. | Unclear to what extent and how exactly monitoring results affect redesigning the measures. | No directly, but a need for new measures has been identified. |
| Sweden | AES-CAP schemes are updated as required within the 7-year CAP period. | Yes (partly) but spatial coverage of monitoring data is poor. | No evidence available. |

## Is there public availability of evaluation processes, and any other uses of monitoring information (including in policy)?

Table 3‑4 WFD: Is there public availability of evaluation processes, and any other uses of monitoring information (including in policy)?

|  | **Are there reports that synthesise and interpret the data available?** | **Are the policy evaluation processes documented?** | **Is there information on how programme of measures were altered due to monitoring data?** | **Is there information about how monitoring data led to revising policy implementation?** |
| --- | --- | --- | --- | --- |
| Catalonia | Processes of data synthesis and interpretation are available on a public website. | Evaluation reports (including results and methods; PoMs) also are available in the same website. | The process of how PoMs were revised is not publicly available. | The process of how RBMPs were revised is not publicly available. |
| Estonia | To a degree this is available in the respective RBMPs, and implementation reports at EC web page^[[4]](#footnote-4)^. This is not directly accessible from Estonian Ministry of Environment’s website. | Yes, in the official progress report formats. | Not evident from the documents/reports. | Not evident from the documents/ reports. |
| Finland | Numerous mid-level reports are available on the internet evaluation reports. | Evaluation reports available on the internet. | Yes, monitoring data is used to evaluate impact of measures and if measure is not efficient based on data it can be changed. | Monitoring data is used, but information how it is used is no clearly written out. |
| Flanders | Reports are available on the website of the Flanders Environment Agency (VMM) covering both WFD and other ‘water issues’. | Evaluation reports are available on the website. | In some evaluation reports, the use of monitoring reports is clear. | Public info on how water policy changes are available but links to monitoring data that determine changes in action plans are scarce. |
| Hungary | Nothing yet although a new project has been launched that may deliver this. | No evidence. | No evidence available. | No evidence available. |
| Romania | Yearly synthesis reports are available on the web site. | No evidence available. | The revised PoMs are based on the monitoring data. | The law highlights the need to consider the monitoring results but the process itself (remains unclear. |
| Scotland | Water body information is available online, which is to some extent a synthesis. Regular evaluation reports could not be found. | There are classification reports (which are not full evaluation reports). | There is some evidence of cases where the data for PoMs is used. Unclear whether this is true across the board. | In RBMPs there is a section with reference to how they were updated. |
| Slovakia | Evaluation reports available on the website. | Yes, the progress of WFD Implementation strategy in the Slovak Republic, River Basin Management Plan, identification of the problem is documented on website. | Probably not. | Public info on how water policy changes are available but links to monitoring data that determine changes in action plans are scarce. |
| Sweden | The ecological classification of all water bodies in Sweden is publically available. | Progress towards achieving WFD goals is evaluated during each six-year cycle. National environmental objectives are reviewed each year and a more in-depth evaluation is done every four years. These reports are available online from the responsible agencies. | How PoMs have been revised is available online. However, much is based on expert analysis, and therefore lacking in transparency. | Information of how RBMPs have been updated is available via five water agencies. |

Table 3‑5 N2K: Is there public availability of evaluation processes, and any other uses of monitoring information (including in policy)?

|  | **Are there reports that synthesise and interpret the data available?** | **Are the policy evaluation processes documented?** | **Is there information on how site management plans were altered due to monitoring data?** | **Is there information about how monitoring data led to revising the implementation of N2K policy?** |
| --- | --- | --- | --- | --- |
| Catalonia | No reports were found. | There are no official evaluations beyond the mandatory reports on the status of development of the N2K network. However, the overall system of protected areas in Catalonia, including N2K sites, was evaluated by the Catalan Institution for natural History (ICHN). | No as site management plans are not yet completed. | No as the policy is still being developed. |
| Estonia | No reports were found. | No reports were found. | No evidence was found - public participation is not required by law for SMP. | No evidence was found. |
| Finland | Yes, there are regular analyses and syntheses available. | There is a formal evaluation procedure of the overall policy. | There is often a lag between the data showing problems and remedial management actions being adopted; and the process remains unclear. | There is a formal evaluation procedure of the overall policy and these reports are based on monitoring data. |
| Flanders | Midlevel reports are available on the website of INBO. | Habitat quality and quantity are evaluated and reported in the public domain. | There is no evidence that monitoring led to changes in site management plans. | Public info on how N2K policy changes or has changed is scarce and not clearly related to N2K monitoring. |
| Hungary | The data synthesis and interpretation of N2K is not standardised, although there are a few useful publications on evaluation. | No evidence found. | No documented evidence available. | No documented evidence available. |
| Romania | There are no synthesis reports publicly available. | There are no evaluation reports publicly available. | It is unclear how the data is being used to revise SMPs. | it is unclear how the monitoring information is being used for the implementation of nature protection policy. |
| Scotland | Mid-level reports are not regularly available (the last report dated 2006). | No evidence found. | The process of the M&E feedback to management is available on a feature by feature basis. | There is little information available online on M&E feedback to policy. |
| Slovakia | Yes, there are regular analyses and syntheses, the works are available at the web pages of Ministry of Environment and State Nature Conservancy. | Evaluation reports are available, downloadable in .pdf. | Yes, there is information included in “Conception of the nature conservation in Slovakia” and regular updates. | Yes, there are subsites of the Ministry of Environment and State Nature Conservancy available for public including such information. |
| Sweden | Regular analyses are available. Coordinated by the Swedish Species Information Centre. | No information. | No information. | No information. |

Table 3‑6 AES: Is there public availability of evaluation processes, and any other uses of monitoring information (including in policy)?

|  | **Are there reports that synthesise and interpret the data available?** | **Are the policy evaluation processes documented?** | **Is there information on how AES measures were altered due to monitoring data?** | **Is there information about how monitoring data led to revising the Rural Development Programme?** |
| --- | --- | --- | --- | --- |
| Catalonia | No ‘mid-level’ or evaluation reports are available. | There are no official evaluation reports of AES in Catalonia. There only are some evaluation reports performed by independent NGO’s proposing improving measures of AES. | No evidence available. | No evidence available. |
| Estonia | General information as well as the most of “raw data” on monitoring results are available, however no ‘mid-level’ or evaluation reports. | Evaluation reports are available | There is no specific info on processes how AES are redesigned | ERDP annual implementation reports contain some general info on recommendations for future change. |
| Finland | Analysis of Data collected in the MaaMet –monitoring programme are available on line. | Programme results and impacts have indicators and metrics^[[5]](#footnote-5)^, but how this impacts on policy is not clear. | The same www-page contains information that helps to understand how national policy has or will be redesigned. | No evidence available. |
| Flanders | For a limited number of AES packages, mid-level reports are available. | For a limited number of AES packages, mid-level and evaluation reports are available. | There is no info that describes the process and how monitoring is used for this feedback, but when the new AES packages launched (± 2015) it was suggested that new AES schemes rely on results of former monitoring. | No public info on how AES policy changes or has changed was found. Changes were made but not clearly related to AES monitoring (although it could be so). |
| Hungary | No ‘mid-level’ report s available. | Monitoring and evaluation processes by MME projects are well published. | The need for monitoring (feedback) is noted in the Rural Development Plan as a necessity in the future, but does not occur at present. | No response received. |
| Romania | Reports focussed on financial features of measures, surfaces covered by the measures, performance achieved. | Evaluation reports are available for RDP, but limited for AES. Annual progress report on program implementation is completed by evaluation (annex comprising questions, evaluation report, conclusions and recommendations). Not always a clear link with Monitoring data. | Not clear what monitoring sources and data are used for redesign AES. | Public have access to AES modified documents. But no monitoring data that led to changes is available. |
| Scotland | There is no access to synthesis of primary data collected by RPID from the inspections. | Ex-post evaluation reports exist but these are infrequent. | No info on the process through which these evaluation or inspection reports, feed back into redesigning AESs or allocating resources. | There is no info on how any aspect of SRDP policy is redesigned using the monitoring data. |
| Slovakia | Annual reports and mid-terms reports, as well as some other statistics are publicly available. | Evaluation reports are available on webpage. | New measures were added due to secondary monitoring data (e.g. birds protection). | No response received. |
| Sweden | Annual reports produced by SEPA (Scottish Environment Protection Agency) summarize fulfilment of national environmental objectives related to agriculture. | Yes, as progress towards achieving national environmental objectives. | No response received. | No response received. |

1. Birdlife Finland website: <https://www.birdlife.fi/in-english/>. [↑](#footnote-ref-1)
2. According to regulation (CE) 1303/2013 articles 47-49 the Monitoring Committee (MC) streamlines data sharing for National Rural Development Program <http://eur-lex.europa.eu/legal-content/en/TXT/?uri=celex%3A32013R1303>) [↑](#footnote-ref-2)
3. For Romania: [www.madr.ro/.../implementare-pndr-2014-2020/comitet-monitorizare.html](http://www.madr.ro/.../implementare-pndr-2014-2020/comitet-monitorizare.html) [↑](#footnote-ref-3)
4. Estonian WFD implementation report from EC website: <http://ec.europa.eu/environment/water/water-framework/impl_reports.htm> . [↑](#footnote-ref-4)
5. See <https://www.maaseutu.fi/en/the-rural-network/program-results/> [↑](#footnote-ref-5)
